# The potential of microbiome modulators to improve quality of life in cancer survivors through gut microbiome-immune interactions

**DOI:** 10.3389/fnmol.2026.1815079

**Published:** 2026-08-27

**Authors:** Brandi C. Miller, Julia H. Hoover, Hariom Yadav, Shalini Jain

**Affiliations:** 1 USF Center for Microbiome Research, Microbiomes Institute, University of South Florida Morsani College of Medicine, Tampa, FL, United States; 2 Center for Excellence in Aging and Brain Repair, University of South Florida Morsani College of Medicine, Tampa, FL, United States; 3 Department of Neurosurgery and Brain Repair, University of South Florida Morsani College of Medicine, Tampa, FL, United States

**Keywords:** brain, cancer survivors, cancer treatments, cognitive impairment, gut, gut microbiome, microbiome-immune axis, quality of life

## Abstract

Advancements in oncology research have led to the development of highly effective and safe cancer treatments, resulting in increased survival rates from cancer. However, treatments including chemotherapy, radiation, immunotherapy, and combination regimens suppress or modulate the immune system, contributing to the development of multi-organ dysfunctions in cancer survivors. Cognitive impairment (CI) and bowel dysfunctions are adverse outcomes commonly reported in cancer survivors and collectively hamper quality of life (QoL). Therefore, it is critical to develop strategies to improve or protect from these side effects and improve QoL in cancer survivors. The gut microbiome is a superlative regulator of human health and abnormalities in its composition and function (gut dysbiosis) contribute to many disease pathologies. Emerging studies suggest that cancer treatments significantly perturb the gut microbiota composition, impacting several peripheral organs, including the gut and brain. Gut dysbiosis also impacts the immune system, suggesting that cancer treatment-mediated gut microbiome alterations can alter brain health through the gut microbiome-immune axis. Thus, beneficial modulation of the microbiome through probiotics, prebiotics, and other interventions may be a feasible approach for improving cognitive function, gut health, and/or QoL in cancer survivors via gut microbiome-immune interactions. The purpose of this review is to report the prevalence of and mechanisms contributing to CI and gut dysfunctions in cancer survivors as well as provide a comprehensive review of the potential of microbiome modulators for improving gut and brain health and QoL in these cohorts, through the gut microbiome-immune axis. Additional studies are warranted to fully elucidate the effects and mechanisms of microbiome modulators in cancer survivors.

## 1 Introduction

The development of highly effective and safer cancer treatments has led to an increase in cancer survival rates in the past few decades. Implementation of modern screening and detection methods has also contributed to increased survival from cancer. According to the 2024 statistics of The National Cancer Society, the average 5-year survival rate from cancer has increased to higher than 69%, with prostate, breast, testicular, and thyroid cancers having some of the highest survival rates (97, 91, 95, and 99%, respectively) (Siegel et al., 2024). Conventional cancer treatments like chemotherapy and radiation effectively remove or reduce tumor burden and disease progression. However, their cytotoxic effects extend past the tumor site and can suppress the immune system and impact peripheral organs such as the gut and brain. Immune system suppression can weaken its response to cancer, increase inflammation, and lead to the development of inflammatory- or immune system-related conditions and infections. Thus, immunotherapies like immune checkpoint inhibitors (ICIs), monoclonal antibodies, and chimeric antigen receptor T-cell (CAR-T) therapy or adjuvants to chemotherapy have been designed to mitigate cancer without suppressing the immune system. Nonetheless, conventional treatments as well as immunotherapies have been linked with inflammation and other adverse outcomes in cancer survivors, including cognitive impairment (CI) and abnormal gastrointestinal (GI) functions.

CI is very commonly reported by cancer survivors and includes deficits in learning and memory, executive functioning, and psychiatric health. CI is most studied in breast cancer survivors, with up to one-third of breast cancer survivors experiencing some degree of CI (Whittaker et al., 2022). Emerging studies also suggest that CI is prevalent in survivors of other cancer types, including colon/colorectal, testicular, prostate, ovarian, and head and neck cancers (Országhová et al., 2021). Furthermore, patients with cancer experience impairments in GI health, especially during treatment, but these symptoms can persist long-term, especially if unmanaged. Constipation, diarrhea, and abdominal bloating or pain are the most common GI symptoms reported in mixed cohorts of cancer survivors (Rietveld et al., 2019; Cherwin and Perkhounkova, 2017; Derogar and Lagergren, 2012; Jiang et al., 2017). Both CI and dysfunctions in GI health significantly reduce quality of life (QoL) in cancer survivors. Decreased QoL further impacts gut and brain functions and can also deteriorate physical and social functions, which collectively hinder day-to-day activities and place substantial social and economic burdens on cancer survivors and their families (Van Leeuwen et al., 2018). There is a critical need for the development of novel and highly effective therapeutics to improve gut and brain functions and overall QoL in cancer survivors. However, this requires a better understanding of the effects and mechanisms of cancer treatments and their mechanisms, which remain elusive.

The gut microbiome, including bacterial, viral, archaeal, fungal, and eukaryotic components, and its metabolites are superlative regulators of human health (Thursby and Juge, 2017). Chronic perturbations in its composition and functions (gut dysbiosis) have been linked with metabolic syndromes (i.e., obesity and diabetes) (Mishra et al., 2019; Turnbaugh et al., 2006), cardiovascular diseases (Tang et al., 2013), CI (Chaudhari et al., 2023; Mishra et al., 2024), and neurodegenerative conditions like Alzheimer’s disease (AD) (Nagpal et al., 2019; Vogt et al., 2017). The gut microbiome is easily modifiable by many factors, including cancer treatments. Emerging studies report that cancer treatments are associated with lower α-diversity, enrichment of potentially inflammatory microbes like Bacteroidetes and Proteobacteria, and deplete beneficial bacteria such as those belonging to phylum Firmicutes (Li et al., 2017; Sougiannis et al., 2019; Montassier et al., 2015; Deleemans et al., 2022a; Chua et al., 2017). Diet also significantly shapes the gut microbiome community and fiber-rich or ketogenic diets, fermented foods, and supplements like probiotics (live bacteria) and prebiotics (indigestible fibers) may improve gut dysbiosis and cognitive health in cancer survivors (Nagpal et al., 2019; Liu et al., 2023; Kim et al., 2021; Ahmadi et al., 2020; Miller et al., 2021). However, their efficacy and widespread use in these cohorts have not been established.

The gut microbiome modulates the gut-brain axis, which is the bidirectional connection between the gut and the brain and contributes to the development of aging-related, neurodegenerative, and psychiatric diseases. Modulation of the gut-brain axis also changes gut microbiome structure and function. The gut and brain are connected physically via the vagus nerve but also communicate via the immune system, microbial metabolites (i.e., short-chain fatty acids [SCFAs] and bile acids), neurons and neurotransmitters, and hormones (Cryan et al., 2019). Thus, the gut-brain axis impacts immunity, inflammation, and oxidative stress, bacterial metabolism, and integrity of the intestinal epithelium and blood–brain barrier (Carabotti et al., 2015). The gut microbiota are also in close proximity with mucosal immune cells and are important for shaping their repertoire and function. They are involved directly in educating and activating innate and adaptive immune cells in response to pathogens, but also indirectly modulate the immune system through the production of SCFAs. Beneficial modulation of the microbiome may decrease AD pathologies or improve psychiatric health through production of anti-inflammatory cytokines or metabolites like SCFAs and restoration of gut and brain barrier integrity (Prajapati et al., 2025b; Yang et al., 2022; Yang et al., 2019). However, such reports are limited, especially in humans. These studies are necessary to fully elucidate the impacts of microbiome modulators on brain and gut health and gut-brain connections in cancer survivors.

The purpose of this manuscript is to review the current literature reporting the effects of cancer treatments on gut and brain health, their impacts on QoL in cancer survivors, and highlight the gut microbiome-immune axis as a major mechanism. We will then focus on the role of the gut microbiome in the development of cancer treatment-related side effects and review current interventional studies investigating the effects of microbiome modulators in cancer survivors.

## 2 Literature selection criteria

In this manuscript, we review the literature regarding (1) the effects of cancer treatments on gut and brain health and QoL in cancer survivors and (2) the potential of microbiome modulators in this population. We used PubMed and Google Scholar to find relevant literature and clinical studies from the past 31 years (1995 to present). Key search terms included “cancer survivors,” “gut,” “gut microbiome,” “cognitive function,” and “quality of life.” To find intervention studies, we included key terms like “intervention,” “probiotics,” “prebiotics,” “synbiotics,” and “fermented foods.” We also searched ClinicalTrials.gov to find ongoing and past registered clinical trials, using inquiries such as “cancer survivor” and included “probiotics,” “prebiotics,” “synbiotics,” and “fermented foods” as intervention key terms. There were no specific criteria regarding cancer type or severity or treatment regimen and we included studies which reported gut-brain or QoL impairments in survivors of any type of cancer who had undergone any treatment for their cancer. We only included studies recruiting adults 18 and older; thus, studies involving cancer survivorship in children were excluded. Studies enrolling adults with a history of pediatric cancer were considered. For both literature reviews, we excluded studies which assessed outcomes exclusively during active cancer treatment. Thus, our focus is on cancer survivorship, including impairments in gut-brain health as a potential result of cancer treatments, and how gut microbiome interventions may improve these impairments and QoL in survivors.

## 3 Use of cancer treatments is linked with side effects in cancer survivors

By 2040, it is predicted that the number of cancer survivors will reach 26 million in the United States alone (Tonorezos et al., 2024). This continual growth in the cancer survivor population can largely be attributed to more efficient diagnostic and screening tools, leading to an earlier diagnosis of cancer, as well as the development of highly effective treatments. Cancer treatments are cytotoxic to both tumors and healthy cells. Their effects can be local and systemic, impacting peripheral organs, which contributes to multiple adverse events and a significantly lower QoL in cancer survivors. Some of the most prevalent effects of cancer treatments include fatigue, nausea/vomiting, hair loss, changes in appetite, and brain fog (“chemobrain”). However, these side effects occur on an individual basis and vary from person-to-person depending on cancer stage and type, treatment type and dosing regimen, and other factors like age, gender, and cancer history. For example, surgery and radiation are often the first-line treatments for breast cancer (Trayes and Cokenakes, 2021), while surgery and systemic chemotherapy or hormone therapy are common for colorectal cancer (CRC) (Hernandez Dominguez et al., 2023). Immunotherapies, such as ICI, monoclonal antibodies, and CAR-T therapy, are becoming increasingly used for treatment of multiple cancers due to increased response by patients with cancer (Jian et al., 2025). Because they modulate the immune system, these treatments may also contribute to the development of adverse effects in survivors, who already have weakened immune systems as a result of cancer treatments, especially when used in combination with chemotherapy (Emens and Middleton, 2015). However, we lack a full understanding of cancer treatment-related side effects, including their prevalence, duration, and mechanisms.

Impairments in cognitive and psychiatric functions and abnormalities in GI health are some of the major adverse effects associated with cancer treatments (Jiang et al., 2017; Rietveld et al., 2019). First, we discuss the prevalence of CI and brain dysfunctions, which has been most reported in breast cancer survivors, but also is prevalent in survivors of other cancer types.

### 3.1 Cognitive impairment and other mental health or psychiatric symptoms

CI is common during aging and presents in many neurodegenerative diseases like AD and other forms of dementia as well as comorbidities like diabetes. CI is defined by changes in cognitive functions, including learning and memory, executive functioning, perception, and language, which all interfere with activities of daily living. In the clinic, CI is measured using well-validated cognitive function questionnaires, including the Montreal Cognitive Assessment (MoCA) (Nasreddine et al., 2005), Mini-Mental State Examination (MMSE) (Arevalo-Rodriguez et al., 2021), and Mini-Cog (Borson et al., 2000), which provide comprehensive cognitive health data when given together. Alterations in psychiatric health (i.e., depression and anxiety) impact more than 30% of patients with cancer and/or survivors. Psychiatric health is evaluated using questionnaires like the Patient Health Questionnaire-9 (PHQ-9; anxiety and depression) (Kroenke et al., 2009), Generalized Anxiety Disorder-7 (Spitzer et al., 2006), Depression Anxiety and Stress Scales-21 (Norton, 2007), and the Functional Assessment of Cancer Therapy (FACT) questionnaire, which is specific for cancer (Costa et al., 2018). Magnetic resonance imaging (MRI), positron emission tomography, and electroencephalography (EEG) record brain activities and structure, which can be correlated with changes in cognitive and psychiatric functions (Walters et al., 2025; Zandifar et al., 2020; Silverman et al., 2008). Implementation of multiple screening and diagnostic tools allows for a comprehensive evaluation of cognitive dysfunctions, which aids the development of potential therapeutic strategies for improving brain health. There are several factors which contribute to CI, including (1) age, (2) pathological changes in the brain (i.e., accumulation of neurotoxic peptides like amyloid-β [Aβ], hyperphosphorylated tau, and α-synuclein), which result in neurodegeneration or changes to brain vasculature, (3) development of chronic diseases, (4) genetic predisposition, and (5) lifestyle factors like smoking or low exercise.

CI is one of the most common symptoms reported in cancer survivors, with up to 75% of survivors experiencing some degree of CI. Short-term CI (3 weeks to one year post-treatment) may be reversible with proper management. However, these pathologies become neurotoxic when chronic and can lead to progressive and irreversible neurodegeneration, resulting in long-term CI (1 year to more than 20 years post-treatment) in cancer survivors (Országhová et al., 2021). Furthermore, cancer treatments double the risk for the development and progression of mental and psychiatric disorders like anxiety and depression (Fernando et al., 2023; Naughton and Weaver, 2014). Changes in cognitive function and psychiatric health significantly lower QoL in cancer survivors. A recent meta-analysis including 147 reports enrolling over 135,000 cancer survivors from 30 countries reported that anxiety, depression, and sleep problems were present in over 20% of the selected studies (Ge et al., 2025). Cancer treatment-related CI (CTCI) has been most studied in survivors of breast cancer, which accounts for ~15% of all diagnoses and has a five-year survival rate of 91% (Siegel et al., 2024). CTCI has also been reported in survivors of colon/colorectal, testicular, gynecologic, prostate, head and neck, and hematologic cancers (Siegel et al., 2024; Rovito et al., 2025). Table 1 shows the prevalence of CTCI and other mental or psychiatric health symptoms in specialized cohorts of cancer survivors.

**Table 1:** Prevalence of CI and other neurocognitive or psychosocial impairments in cancer survivors.

Year	Study cohort	Treatment(s)	Prevalence of CI
Breast cancer			
van Dam et al. (1998)	1. 70 breast cancer survivors (≥ 2 years post-treatment) 2. 34 controls Mean age: 46.1 years	1. High-dose FEC chemotherapy + tamoxifen (*n* = 34, mean age: 45.5 years) 2. Standard dose FEC chemotherapy plus tamoxifen (*n* = 36, mean age: 48.1 years)	32% with CI (high dose cohort) 17% with CI (standard dose cohort) 9% with CI (controls) 8.2-and 3.5-times higher risk of CI, respectively, in high dose and standard dose groups, compared to controls
Wefel et al. (2004)	1. 18 survivors of non-metastatic breast carcinoma Mean age: 45.4 years	1. 5-FU, doxorubicin, cyclophosphamide chemotherapy + methotrexate/vinblastine	61% with CI (3 weeks post-treatment) 25% with CI (1 year post-treatment)
Castellon et al. (2004)	1. 53 breast cancer survivors (2-5 years post diagnosis or surgery) 2. 19 non-cancer controls Mean age: 49.2 years	1. Surgery + chemotherapy +/− tamoxifen (n = 36, mean age: 46.8 years) 2. Surgery + local therapy only (n = 17, mean age: 48.3 years)	↓ verbal learning, visuospatial functioning, and visual memory (chemotherapy cohort) ↑ CI in adjuvant chemotherapy + tamoxifen cohort
Hurria et al. (2006)	1. 45 survivors of stage I, II, or III breast cancer Mean age: 70 years	1. Adjuvant chemotherapy (chosen on patient-to-patient basis)	51% with CI (6 months post-treatment)
Koppelmans et al. (2012)	1. 196 breast cancer survivors Mean age: 64.1 years 2. 1,509 non-cancer controls Mean age: 57.9 years	1. Adjuvant CMF chemotherapy (21 years prior, on average)	↓ immediate and delayed verbal memory, processing speed, executive functioning, and psychomotor speed (chemotherapy cohort)
Von Ah et al. (2013)	1. 22 breast cancer survivors Mean age: 56.1 years	1. Surgery and/or chemotherapy (1-12 years prior)	↓ short- and long-term memory, processing speed, attention and concentration, language, and executive function
Buchanan et al. (2015)	1. 2,296 breast cancer survivors (≥ one year post-treatment) Age range: 28-78 years	1. Chemotherapy (*n* = 288) 2. Hormone therapy (*n* = 822) 3. Chemotherapy + hormone therapy (*n* = 859) 4. Neither (*n* = 327)	60% with self-reported cognitive problems ↑ cognitive complaints with chemotherapy, hormone therapy, or both, compared to “neither” group
Janelsins et al. (2017)	1. 581 breast cancer survivors Mean age: 53.1 years 2. 364 controls Mean age: 53 years	1. Chemotherapy (followed by radiation or hormone therapy up to 6 months post-treatment)	45.2% with CI (post-chemotherapy) 36.5% with CI (6 months post-chemotherapy)
Van Dyk et al. (2017)	1. 103 breast cancer survivors Mean age: 56.8 years	1. Radiation (*n* = 32) 2. Chemotherapy (*n* = 11) 3. Chemotherapy + radiation (*n* = 45) 4. Neither (*n* = 15)	↓ perceived cognition, perceived CI, and QoL domains in low-memory performing and depressed survivors on FACT-Cog
Boscher et al. (2020)	1. 1393 breast cancer survivors Median age: 52 years	1. Chemotherapy (*n* = 1065) 2. Endocrine therapy (*n* = 1039) 3. Targeted therapy (*n* = 133)	33.9% with anxiety 9.4% with depression symptoms 8.3% with fatigue 27.7% with post-traumatic stress 47.2% with cognitive complaints
Carreira et al. (2021)	1. 356 breast cancer survivors Mean age: 64.8 years 2. 252 non-cancer controls Mean age: 65.5 years	1. Surgery (35%) 2. Radiotherapy (80%) 3. Hormone therapy (49%) 4. Chemotherapy (41%)	↓ cognitive-related QoL ↑ risk of anxiety symptoms Advanced cancer stage and chemotherapy linked with poorer QoL
Alwi et al. (2021)	1. 160 early-stage breast cancer survivors Mean age: 51.5 years	1. Chemotherapy < 24-month post-treatment > 24-month post-treatment	31.9%, 53.8%, and 51.3% of cohort performed poorly on MoCA, Rey AVLT, and WMI of WAIS-IV, respectively No significant differences between <24 month and >24 month groups
Carreira et al. (2021)	1. 335 breast cancer survivors Mean age: 63.9 years	1. Chemotherapy followed by surgery and/or radiation	18.6% with CI in at least one domain 26% reported poor to moderate subjective attentional function
Von Ah et al. (2022)	1. 498 breast cancer survivors Mean age: 45.3 years 2. 394 healthy controls Mean age: 46.5 years	1. Chemotherapy followed by radiation (69%) 2. Chemotherapy followed by hormonal therapy (39.4%)	23% of survivors with memory problems, significantly higher than controls
Luo et al. (2023)	1. 404 breast cancer survivors Mean age: 70.4 years 2. 2016 controls Mean age: 70.3 years	1. Not specified	↑ CI prevalence following cancer diagnosis (20+ years later) Accelerated CI in women ≥80 years or with advanced cancer
Kerkmann et al. (2025)	1. 53 breast cancer survivors	1. Chemotherapy *n* = 24, median age: 50 years 2. No chemotherapy *n* = 29, median age: 54 years	↓ cognitive function, figural memory, and QoL in chemotherapy group (within 6 months) ↑ neurodegenerative markers (i.e., NfL and pNfH) with chemotherapy Cognitive function QoL stabilized within 2-3 year follow-up
Jose et al. (2026)	1. 79 women with breast cancer Age range: 20-60 years Pre-chemotherapy (*n* = 25) Post-chemotherapy (*n* = 27) Survivors (*n* = 27)	1. Adjuvant or neoadjuvant chemotherapy	↓ cognitive function and memory in survivors and post-chemotherapy patients compared to pre-chemotherapy controls
Colon/colorectal cancer			
Cruzado et al. (2014)	1. 81 colon cancer patients Mean age: 66.96 years	1. FOLFOX4 (up to 6 months duration)	Verbal memory decline post-FOLFOX4 (56%) Worsening in at least one test 6-months post FOLFOX4 (33%)
Vardy et al. (2015)	1. 362 CRC survivors Localized CRC: *n* = 289 Metastatic/recurrent CRC: *n* = 73 Median age: 59 years	1. Surgery and/or adjuvant or neoadjuvant chemotherapy (FU, oxaliplatin) and/or chemoradiation	43% with CI (at baseline) 46% of survivors with CI (12 months post-treatment) Attention/working memory, verbal learning/memory, and complex processing speed most affected
Liu et al. (2022)	1. 29 CRC survivors Mean age: 58.2 years 2. 29 age-matched controls Mean age: 56.97 years	1. CAPOX chemotherapy +/− bevacizumab	↓ brain activity on MRI post-chemotherapy Brain activity was positively correlated with cognitive function
Yang H. Y. et al. (2023) and Yang J. et al. (2023)	1. 63 CRC patients or survivors New diagnoses (*n* = 13, mean age: 62.15 years) Survivors (<2 years post-treatment, *n* = 24, mean age: 57.0 years) Survivors (>2 years post-treatment, *n* = 26, mean age: 58.1 years)	1. Adjuvant chemotherapy	No significant differences in cognitive function and objective performance ↑ reaction times in attention and processing in CRC survivors <2 years post-chemotherapy
Testicular cancer			
Schagen et al. (2008)	1. 182 testicular cancer survivors Median time since treatment: 3 years	1. Surgery + BEP chemotherapy (Cohort 1, *n* = 70, mean age: 32.1 years) 2. Surgery + radiation (Cohort 2, *n* = 57, mean age: 38.9 years) 3. Surgery only (Cohort 3, *n* = 55, mean age: 34.4 years)	32%, 32%, and 27% of patients in cohorts 1, 2, and 3, respectively, reported cognitive dysfunction Cognitive deficits linked with emotional distress and fatigue
Wefel et al. (2014)	1. 69 testicular cancer survivors Mean age: 31.0 years	1. Adjuvant chemotherapy (*n* = 55) 2. No adjuvant chemotherapy/other (*n* = 14)	52% with CI (low-exposure group) compared to 67% (high-exposure group) after 12 months ↓ motor dexterity in low and high exposure groups (48% and 46%, respectively)
Stouten-Kemperman et al. (2015)	1. 51 testicular cancer survivors	1. Orchidectomy + BEP chemotherapy (*n* = 28, mean age: 43.1 years) 2. Orchidectomy only (*n* = 23, mean age: 48.2 years)	↓ cognitive function and ↑ memory problems in surgery + chemotherapy group, compared to surgery alone
Amidi et al. (2015)	1. 72 testicular cancer survivors Mean age: 40.1 years	1. Orchidectomy + BEP chemotherapy (*n* = 36) 2. Orchidectomy +/− radiation (*n* = 36)	62.5% with CI Significant CI observed on 75% of cognitive function measures
Chovanec et al. (2018)	1. 155 testicular cancer survivors Median age: 41 years	1. Orchidectomy (control, *n* = 17) 2. Orchidectomy + radiation (*n* = 12) 3. Orchidectomy + adjuvant radiotherapy (*n* = 12) 4. Orchidectomy + therapeutic radiotherapy (*n* = 4) 5. Orchidectomy + chemotherapy (*n* = 119) 6. Orchidectomy + chemotherapy + radiation (*n* = 7)	↓ cognitive function in surgery + treatment groups compared to surgery alone ↓ overall cognitive function, perceived CI, and CI perceived by others (radiation only groups) Chemotherapy + radiation or radiation groups: impairment in all cognitive domains
Gynecologic cancer			
Hess et al. (2015)	1. 231 patients with ovarian, peritoneal, or fallopian tube cancer	1. Chemotherapy, 6 cycles i.v., *n* = 172 i.p., *n* = 59	21.1% exhibited CI after 6 cycles of chemotherapy 17.8% exhibited CI in at least one domain at the 6-month follow-up
Prostate cancer			
Gonzalez et al. (2015)	1. 142 prostate cancer patients 2. 88 age- and education-matched healthy controls Mean age: 69.1 years	1. ADT (*n* = 58, mean age: 67.31 years) 2. Prostatectomy (*n* = 84, mean age: 67.72 years)	↑ CI at 6- and 12-month follow-ups in ADT group
Holtfrerich et al. (2020)	1. 24 prostate cancer patients Mean age: 68.67 years 2. 22 prostate cancer controls Mean age: 67.05 years 3. 22 healthy controls Mean age: 65.73 years	1. ADT (up to 20 years post-treatment)	Long-term ADT was linked with ↓ cognitive function ADT was positively associated with depression symptoms ADT was negatively linked with QoL
Head and neck cancer			
Zer et al. (2018)	1. 80 survivors of head and neck cancer Mean age: 58.3 years 2. 40 healthy controls Mean age: 54.6 years	1. Radiation only (*n* = 11) 2. Radiation + cisplatin (*n* = 49) 3. Radiation + carboplatin (+/− FU, *n* = 2) 4. Radiation + panitumumab (*n* = 18)	38% experienced CI 24-months post-treatment compared to controls (0%) ↓ intellectual capacity, concentration, short-term attention, verbal memory, executive function, and global cognitive function over time in survivors
Pruijssen et al. (2022)	1. 29 survivors of head and neck neoplasms Median age: 41 years	1. External beam radiation (*n* = 29) 2. Radiation + chemotherapy (*n* = 10) 3. Radiation + surgery (*n* = 29)	↓ episodic memory and ↑ fatigue in survivors 44.8% with fear of tumor reoccurrence or speech deficits
Skin cancer			
Rogiers et al. (2020a)	1. 25 survivors of metastatic melanoma Median age: 58 years	1. Pembrolizumab (at least 6 months prior)	↓ global health-related QoL 64% experienced clinical anxiety/depression 48% with cancer-related post-traumatic stress disorder, with 28% developing transient suicidal ideation 32% reported CI
Rogiers et al. (2020b)	1. 17 melanoma survivors Median age: 57 years	1. Ipilimumab	Reported (1) fear of recurrence, (2) existential problems, (3) survivor guilt, and (4) post-traumatic stress disorder 41% of survivors with neurocognitive impairment Anxiety or depression reported (*n* = 9)
Boekhout et al. (2021)	1. 89 advanced melanoma survivors 2. 265 controls	1. Ipilimumab	↓ cognitive and social functioning (survivors)
Hematologic cancers			
Barata et al. (2022)	1. 118 patients with non-Hodgkin’s lymphoma Mean age: 61 years	1. CAR-T therapy 85% axicabtagene ciloleucel 13% tisagenlecleucel 2% brexucabtagene autoleucel	↓ perceived cognition from days 90 to 360 ↓ memory, language, organization, and divided attention
Hoogland et al. (2022)	1. 117 patients with non-Hodgkin’s lymphoma Mean age: 61 years	1. CAR-T therapy 87% received axicabtagene ciloleucel	↓ total neurocognitive performance and executive functioning at day 90 ↓ visuospatial abilities over time
Ho et al. (2025)	1. 1407 survivors of hematologic cancers across 16 studies	1. CAR-T therapy	Pooled prevalence of CTCI highly variable across studies 24% (< 1 month post-treatment) 33% (1-12 months post-treatment) 35% (> 12 months post-treatment)
Mixed survivor cohorts			
Vanlaer et al. (2024)	1. 70 survivors of any type of unresectable stage III/IV cancer Median age: 65 years	1. Immune checkpoint blockade therapy	54.3% of survivors experienced clinical fear of cancer recurrence ↑ cognitive complaints were present in 18.6% of survivors
Candido et al. (2025)	1. 132 survivors of melanoma, non-small cell lung cancer, urothelial cell carcinoma, or renal cell carcinoma Mean age: 65 years 2. 80 caregivers of patients with cancer	1. ICIs	28% of survivors with low QoL 17% of survivors with neurocognitive concerns 15% with formally evaluated CI Depression and anxiety significantly correlated with QoL
(Thong et al., 2025)	1. 6057 long-term survivors of breast, colorectal, or prostate cancers Mean age: 69 years 2. 1953 controls Mean age: 55.6 years	1. Chemotherapy 2. Radiotherapy 3. Hormone therapy	~1/3 of survivors reported affective, cognitive, or physical fatigue Age, relationship status, chemotherapy use, depression, lifestyle, and psychological factors were linked with higher risk of physical and total fatigue

ADT: Androgen deprivation therapy, AVLT: Auditory Verbal Learning Test, BEP: Bleomycin, etoposide, and cisplatin chemotherapy, CAPOX: Capecitabine + oxaliplatin chemotherapy, CAR-T: Chimeric antigen receptor-T cell therapy, CI: Cognitive impairment, CMF: Cyclophosphamide/methotrexate/fluorouracil chemotherapy, CRC: Colorectal cancer, CTCI: Cancer treatment-related cognitive impairment, FACT-Cog: Functional Assessment of Cancer Therapy – Cognitive Function, FEC: Fluorouracil, epidoxorubicin, and cyclophosphamide, FOLFOX4: 5-fluorouracil/leucovorin chemotherapy, FU: Fluorouracil, ICI: Immune checkpoint inhibitors, i.p.: Intraperitoneal, i.v., Intravenous, MoCA: Montreal Cognitive Assessment, MRI: Magnetic resonance imaging, NfL: Neurofilament light chain, pNfH: phosphorylated Neurofilament heavy chain, QoL: Quality of life, WAIS: Wechsler Adult Intelligence Scale, WMI: Working memory index, 5-FU: 5-fluorouracil.

Early studies reported that 25–50% of breast cancer survivors experienced some degree of CI, including deterioration of verbal and visual learning, short- and long-term memory, processing speed, attention and concentration and executive function, within 6-months to 1 year post treatment (Wefel et al., 2004; Hurria et al., 2006; Von Ah et al., 2013; Castellon et al., 2004). Recent studies also show short- and long-term impairments in memory and cognitive function post-chemotherapy (Jose et al., 2026). However, these studies included small sample sizes (*n* ≤ 60), limiting their generalizability, and significant heterogeneity in treatment regimen and time-since-treatment. Larger cohort studies reported that 36–50% of survivors experienced CI or other brain dysfunctions up to 6 months following treatment with mixed regimens (i.e., chemotherapy, endocrine therapy, or combinations) (Janelsins et al., 2017; Boscher et al., 2020). These studies suggest that cancer treatments significantly impact cognitive function in the short-term following treatment in breast cancer survivors. In more long-term studies, CTCI has been reported in 17% to greater than 60% of breast cancer survivors within three years of treatment (van Dam et al., 1998; Buchanan et al., 2015; Alwi et al., 2021). The high range in CI prevalence may be due to different treatments (chemotherapy, chemotherapy + tamoxifen, hormone therapy, and chemotherapy + hormone therapy combinations) or cancer severity. Future studies should address these confounding variables using methods such as multivariate analysis so we can understand the impact of different treatment and/or cancer types individually. Treatment dose also has a significant impact on cognitive function; one study reported that high-dose chemotherapy + tamoxifen resulted in a 15% increase in the prevalence of CI (32%, compared with 17% who received the standard dose) (van Dam et al., 1998). Similarly, 18–25% of survivors experienced significant CI and lowered QoL, 3 to 8 years post-chemotherapy (+/− surgery or radiation), compared to age-matched controls without a history of cancer (Van Dyk et al., 2017; Carreira et al., 2021; Von Ah et al., 2022; Crouch et al., 2022). Chemotherapy alone has been linked with significant impairments in cognitive health and QoL within 6 months of treatment, but these impairments may stabilize to baseline within 2–3 years (Kerkmann et al., 2025). In contrast, long-term CI has also been reported in cancer survivors. Individuals who had completed chemotherapy or other treatments more than 20 years prior experienced significantly worse or accelerated cognitive function, particularly lower verbal memory, processing speed, executive functioning, and psychomotor speed, compared to controls (Koppelmans et al., 2012; Luo et al., 2023). Advanced cancer stage and older age were high risk factors for developing CTCI, suggesting that multiple factors contribute to the development of CI in survivors (Luo et al., 2023). This finding suggests that confounding variables like cancer severity and age may contribute to CTCI development – thus, future studies are critical to determine the sole impact of cancer therapy on cognitive health.

Increasing evidence suggests that CTCI also persists in survivors of colorectal, testicular, ovarian, prostate, and head and neck cancers. Reductions in verbal learning and memory, attention and working memory, and complex processing speed were reported in 33–46% of CRC survivors who had undergone chemotherapy (oxaliplatin plus 5-fluorouracil/leucovorin [FOLFOX4]) up to one year prior (Cruzado et al., 2014; Vardy et al., 2015). Elevated interleukin (IL)-6, IL-8, IL-10, and IL-12 levels were associated with lower information processing speed, suggesting that immune system activation and systemic inflammation were involved (Vardy et al., 2015). A recent study confirms these impairments in attention and processing speed in CRC survivors (< 2 years post-chemotherapy), with no significant differences in overall cognitive function compared to controls (Yang H. Y. et al., 2023). Functional MRI revealed that cognitive changes on the MoCA, MMSE, and FACT-Cog were positively associated with decreased brain activity in the left anterior cingulate gyrus in CRC survivors who had undergone chemotherapy with or without bevacizumab (Liu et al., 2022). This shows the effects of chemotherapy on specific brain regions, especially those susceptible to acute neurotoxicity and which are involved in cognitive processes. Furthermore, in survivors of testicular cancer, lymph node radiation in combination with surgery or cisplatin-based chemotherapy was linked with significantly higher emotional distress and fatigue as well as reduced scores on all domains of the FACT-Cog by 8.7 and 16.5%, respectively, compared to surgery or chemotherapy alone (Chovanec et al., 2018; Schagen et al., 2008). Between one and seven years post-treatment, 50–70% of survivors experienced CI, and higher doses or longer duration of chemotherapy were associated with more significant impacts on cognitive functions and motor dexterity (Wefel et al., 2014; Amidi et al., 2015). This finding was also corroborated in a longer-term (~14-year) follow-up study, which suggested that testicular cancer survivors who underwent chemotherapy (+/− surgery) experienced significantly worse cognitive function compared to surgery alone (Stouten-Kemperman et al., 2015). More white matter hyperintensities (suggesting more neuronal demyelination or ischemic injury) were observed in individuals who had undergone chemotherapy, according to MRI (Stouten-Kemperman et al., 2015). Approximately 18% of ovarian cancer survivors experienced CI in at least one cognitive domain six months post-chemotherapy (Hess et al., 2015). Furthermore, prostate cancer survivors who had received standard androgen-deprivation therapy experienced significant impairments in cognitive performance and increased depression, even up to 20 years post-diagnosis (Gonzalez et al., 2015; Holtfrerich et al., 2020). Chemotherapy, radiotherapy, and chemoradiation were associated with CI in up to 38% of head and neck cancer survivors, but CI was not linked with serum inflammatory markers (i.e., IL-1β, IL-2, IL-4, IL-6, IL-8, tumor necrosis factor-*α* [TNF-α], or interferon-*γ* [IFN-γ]) (Zer et al., 2018). Worsened episodic memory and increased fatigue were reported in a cohort of 29 head and neck cancer survivors and were not linked with changes in white matter or neural atrophy, according to MRI (Pruijssen et al., 2022). Cognitive, affective, and physical fatigue were reported by approximately one-third of long-term (5 + years) survivors of breast, colorectal, or prostate cancer (Thong et al., 2025). Logistic regression analysis revealed that age, relationship status, chemotherapy use, and depression-related symptoms were the top factors associated with physical and total fatigue (Thong et al., 2025).

Immunotherapies, particularly ICIs and CAR-T, have been linked with CI during and several years following treatment. Nearly 20% of a multi-ethnic cancer patient cohort reported significant CI, which was correlated with impaired physical function and increased depression and anxiety symptoms (Sayer et al., 2025). This finding reinforces the significance of CI on other QoL measures. In survivors, ICI therapy was linked with impaired psychosocial health and CI in 20–30% of the cohort up to two years following treatment (Candido et al., 2025). Pembrolizumab use was also linked with increased emotional distress (i.e., post-traumatic stress, anxiety, and depression) and neurocognitive complaints in a small cohort of melanoma survivors (Rogiers et al., 2020a). Another cohort study revealed similar findings – melanoma survivors demonstrated significantly poorer cognitive functions and psychosocial health compared to controls (Boekhout et al., 2021; Rogiers et al., 2020b). Treatment with immune checkpoint blockade therapy was also linked with deficits in neurocognitive and psychosocial health in advanced cancer survivors (Vanlaer et al., 2024). Furthermore, a recent meta-analysis including about 1,400 patients revealed that CI was reported in approximately one-quarter of survivors within one month of CAR-T therapy; about 35% experienced CI at the long-term follow-up (> 12 months) (Ho et al., 2025). In survivors of blood cancers (i.e., non-Hodgkin’s lymphoma) who had undergone CAR-T therapy, total neurocognitive performance and perceived cognition were significantly lower within 3–12 months of treatment (Barata et al., 2022; Hoogland et al., 2022). Together, these studies highlight the prevalence of cognitive and psychosocial complaints in cancer survivors – however, the number of studies are limited. The Cog-Immuno trial is a longitudinal, multi-site study designed to assess cognitive function, brain structure and neuroinflammation, and QoL in the short-term following treatment with immunotherapy (anti-programmed death-1/programmed death ligand-1 [PD-1/PD-L1], anti-cytotoxic T-lymphocyte-associated antigen 4 [CTLA4] monotherapy, or combination regimens) (Lange et al., 2022).

These studies report the prevalence of CI or other psychiatric or mental health deficits in cancer survivors. However, these studies are limited by (1) small sample sizes, (2) heterogeneity in study design, time-since-treatment, treatment type, and cancer type, and (3) differences in outcome measures. Further studies with larger, specialized cohorts and analyses to address confounding variables could provide stronger insight regarding the prevalence and duration of brain health impairments following cancer treatment. Reducing confounding variables or bias will allow for better understanding of the impacts of cancer treatments themselves, since there are many factors, such as age, gender, genetics, cancer history and severity, comorbidities, and lifestyle habits which impact cognitive health (Matthews et al., 2026).

### 3.2 Mechanisms of cognitive impairment in cancer survivors

Although limited, these studies begin to highlight the short- and long-term prevalence of CI in survivors of multiple types of cancer; however, very few provide mechanistic insight. A few studies have used imaging techniques like MRI to observe changes in brain structure, signaling, and detect pathological features. Quantitative EEG has revealed changes in theta AP brain waves, which are highly involved in cognitive processes, in breast cancer survivors experiencing CI (Van Dyk et al., 2017). A recent systematic review revealed that structural changes in brain networks like the central executive and dorsal attention networks are associated with chemotherapy use, which were linked with impairments in multiple cognitive domains (Leskinen et al., 2025). Injury to white cerebral matter (i.e., myelinated axons), damage to microvasculature, blood–brain barrier damage, and dysregulations in myelination and hippocampal neurogenesis are linked with chemotherapy (i.e., methotrexate, cisplatin, or 5-fluorouracil [5-FU]) or CAR-T (Dietrich et al., 2006; Han et al., 2008; Geraghty et al., 2025; Patai et al., 2025; Csik et al., 2025). A few studies presented here reported white matter hyperintensities in testicular and head and neck cancer survivors, suggesting that chemotherapy may increase neural atrophy to glial cells and/or their progenitors. Methotrexate, 5-FU, oxaliplatin, radiation, and CAR-T therapy also have been connected with impairments in hippocampal neurogenesis (Sharpe et al., 2012; Han et al., 2008; Seigers et al., 2008; Wefel et al., 2004; Boscher et al., 2020; Nokia et al., 2012; Rola et al., 2004; Winocur et al., 2006; Geraghty et al., 2025). Transcriptomic changes in post-mortem brain tissue from cancer survivors also reveal similar impairments in microglia and oligodendrocyte function (Whalley, 2025). Both neurogenesis and myelination are intricately involved in neural plasticity and brain function/repair mechanisms, including learning and memory formation (neurogenesis) and effective nerve conduction (myelination). Their dysregulation may be associated with memory and learning deficits and neural atrophy (Shors et al., 2001). Larger cohort studies in cancer survivors as well as *in vivo* studies are essential for strengthening the current evidence linking cancer treatments with CI or psychiatric symptoms and validating their mechanisms. Assessing neurodegenerative markers like Aβ, phosphorylated tau, neurofilament light chain, or phosphorylated neurofilament heavy chain in serum or cerebrospinal fluid could further aid in connecting cancer treatments with CI and determine potential mechanisms (Kerkmann et al., 2025). Combining analyses of serum cytokines and lipids as well as imaging techniques are necessary to deepen our mechanistic understanding of CI in cancer survivors. Other factors like age and cancer severity contribute to brain health and are major risk factors for CI or dementia (Hou et al., 2019). There are several host-specific confounding factors, including age, cancer type and severity, treatment type and duration, genetics, diet and exercise patterns, and other lifestyle choices like smoking and alcohol consumption which impact cognitive function, irrespective of cancer history. Therefore, it is important to conduct additional cohort studies with univariate and/or multivariate analyses to strengthen our understanding of how these factors shape the prevalence of CI in cancer survivors and the sole contribution of cancer therapies. Many studies are limited by small sample sizes, underpinning the importance of conducting larger-scale studies to fully understand the impacts of cancer therapies on gut-brain health. Lastly, there is a lack of longitudinal follow-up studies investigating the impacts of cancer therapies on cognitive and psychiatric functions. Additional longitudinal studies should continue to investigate the long-term impacts of cancer treatments on cognitive function in larger and more diverse cohorts of cancer survivors.

### 3.3 Gastrointestinal symptoms

Chemotherapy (i.e., 5-FU and irinotecan), radiation, immunotherapy (i.e., ICIs), and combination regimens (i.e., FOLFLOX4) are commonly associated with the development of adverse GI symptoms (Rangwala et al., 2012; Sánchez-Lara et al., 2013; Kim et al., 2003; Ardizzoni et al., 2007; Douillard et al., 2010). According to patient-reported questionnaires, diarrhea, increases in bowel urgency and frequency, strain with passing stools, rectal pain or bleeding, and flatulence are the most common GI symptoms burdening cancer survivors both short- and long-term (Muls et al., 2013; Rangwala et al., 2012). Diarrhea is a very common side effect of chemotherapy, immunotherapy, and pelvic radiation, impacting up to 80% of the cancer survivor population, and can progress to colitis, severe dehydration, or electrolyte imbalances, which can be fatal if unmanaged (Andreyev et al., 2012; Du Bois et al., 1992; Larkin et al., 2015; Som et al., 2019; Muls, 2014; O’Reilly et al., 2020).

Preliminary findings from the Chemo-Gut Study reported that over half of survivors (mostly receiving chemotherapy, radiation, hormone therapy, surgery, or immunotherapy) experienced constipation, diarrhea, and bloating or abdominal pain for an average of 30 months post-treatment (Deleemans et al., 2022b; Deleemans et al., 2019). Interestingly, worse GI health was linked with poorer mental and physical health outcomes in this study and in a cohort of ovarian cancer survivors (Deleemans et al., 2022b; Rietveld et al., 2019). Similarly, breast cancer survivors who had received chemotherapy experienced significantly more nausea, diarrhea, flatulence, dry mouth, changes in taste, and poor appetite compared to controls (Hoang et al., 2024). QoL was significantly reduced in at least 20% of gastric cancer survivors who had undergone chemotherapy pre- or post-surgery (at least 12 months prior), mostly owing to fatigue, abnormalities in GI health (dysphagia and diarrhea), and CI (Kim et al., 2012).

QoL was significantly reduced in at least 20% of CRC survivors who had undergone chemotherapy pre- or post-surgery (> 12 months prior), mostly from abnormalities in GI health (McQuade et al., 2014). Strikingly, one study reported that post-treatment diarrhea persisted for up to 10 years after chemotherapy in CRC survivors (Kim et al., 2012). A recent large-scale study in female CRC survivors reported that 81% of the cohort (*n* = 413) experienced persistent perturbations in GI health, even in individuals diagnosed with cancer 8 years prior (Han et al., 2023). Gas/bloating, constipation, diarrhea, and pelvic pain were the most common and severe symptoms reported in this cohort. Furthermore, CRC survivors (62.5% male) experienced flatulence as the most common GI symptom within three years of treatment, but diarrhea and constipation were also prevalent (O’Gorman et al., 2018). Among 137 long-term survivors (≥ 3 years) of pancreatic cancer who had undergone pancreatoduodenectomy, 99% reported some degree of GI dysfunction, including reflux, pancreatic insufficiency, and delayed gastric emptying; however, QoL was not significantly affected (Zhang et al., 2024). Among 191 ovarian cancer survivors, 20% experienced high GI distress, with constipation, pain, nausea/vomiting, and diarrhea being the most prevalent (Rietveld et al., 2019).

These studies collectively show that cancer survivors may experience short- and long-term GI symptoms, with some impacting QoL, but are limited because of (1) small sample sizes, (2) heterogeneity in cancer and/or treatment type, study design, and consistent outcome measures, and (3) lack of long-term follow-up or longitudinal study design. Longitudinal analysis will allow us to better clarify the progression of GI-related symptoms since treatment; currently, there is a lot of heterogeneity in reported GI outcome measures, making it unclear how long GI symptoms persist in cancer survivors. Additionally, some studies have reported no significant GI-related symptoms following cancer treatment, adding complexity to our understanding of if and how cancer treatments impact the gut (Schneider et al., 2007). It is important to consider that these symptoms occur on a patient-to-patient basis and highly vary due to several host-specific and environmental factors, such as genetics, age, cancer type and severity, prevalence of comorbidities, and lifestyle habits. Furthermore, many of the studies report abnormal bowel functions in survivors of CRC and pancreatic cancer and these symptoms may be more prevalent because the cancer is GI-related. Additional studies including multivariate analyses and larger sample sizes are necessary to further disentangle the connections between cancer type, treatment history, and abnormal GI health.

### 3.4 Mechanisms of gastrointestinal symptoms in cancer survivors

Chemotherapy agents like 5-FU, methotrexate, and irinotecan most commonly lead to intestinal mucositis, which is characterized by damage to villus structures, crypt cells, and levels of intestinal enzymes and often culminates in diarrhea (McQuade et al., 2014; Chang et al., 2012; Shen et al., 2021; Boukhettala et al., 2009; Yu et al., 2022). In these studies, structural changes in the intestine were linked with increased myeloperoxidase activity and elevated levels of proinflammatory cytokines, including IL-6, TNF-α, and IL-1β (Shen et al., 2021; Chang et al., 2012). Furthermore, platinum-based agents, such as cisplatin and oxaliplatin, impair the balance of electrolytes (such as magnesium) and water (Ariceta et al., 1997; Oronsky et al., 2017). Both intestinal atrophy and micronutrient imbalances are linked with mucositis, resulting in vomiting, diarrhea, as well as impairments in nutrient absorption, further accelerating the damage. The gut microbiome is also a principal component contributing to gut health and it has a prominent role in cancer pathology. Some studies have described a distinct cancer-associated microbiome- referred to as an “oncobiome”- characterized by an enrichment of *Escherichia*/*Shigella*, *Citrobacter*, *Flavobacterium*, *Acinetobacter*, *Klebsiella*, *Prevotella*, and *Chryseobacterium* and depletion of *Lachnospiraceae*, *Bifidobacterium*, and *Streptococcus* (Tjalsma et al., 2012; Chen et al., 2012; Sobhani et al., 2011). *Fusobacterium nucleatum* and *Clostridium* (*Cs.*) *difficile* have been implicated as major species involved in CRC pathology (Fukugaiti et al., 2015; Zheng et al., 2017). Apart from cancer itself, cancer treatments like 5-FU and methotrexate are associated with significantly lower total bacterial counts, reduced diversity, enrichment of potentially pathogenic aerobes like *Enterococcus* (i.e., *En. faecium*), and decreased abundances of beneficial bacteria like *Lactobacillus*, *Bifidobacterium*, *Veillonella*, and *Faecalibacterium* (*Fb.*) *prausnitzii* (Fijlstra et al., 2015; Nayak et al., 2021; Sougiannis et al., 2019; van Vliet et al., 2009; Zwielehner et al., 2011). Radiation therapy is also linked with decreased gut microbiome diversity, lower abundances of total anaerobes, depletion of Firmicutes, *En. faecalis*, *Lactobacillus* species, and enrichment of Fusobacteria, *Cs. difficile*, and *Cs. perfringens* in gynecological and abdominal cancers (Cuzzolin et al., 1992; Nam et al., 2013; García-Peris et al., 2012; Mitra et al., 2020).

Emerging studies in human cancer survivor cohorts have reported changes in the gut microbiome composition and/or function and linked them with changes in GI functions. Chemotherapy-induced diarrhea was associated with depletion of *Lactobacillus*, *Bifidobacterium*, *Bacteroides*, *Enterococcus*, and methanogenic archaea and enrichment of *Staphylococcus* and *Escherichia* (*E.*) *coli* in a mixed cancer survivor cohort (Stringer et al., 2013). Furthermore, *Prevotella_9*, *Akkermansia*, *Lachnospira*, and *Lachnospiraceae_NK4A136* were able to differentiate breast cancer survivors with GI symptoms like nausea, diarrhea, and flatulence, from age- and sex-matched healthy controls (Hoang et al., 2024). In the Chemo-Gut study, *Lachnospiraceae* was positively correlated with diarrhea occurrence, cognitive health, and anxiety in survivors 6 months post-treatment (Deleemans et al., 2022a). Furthermore, pelvic radiation increased diarrhea occurrence and serum inflammatory markers (i.e., TNF-α), which were associated with depletion of *Clostridium_XIVa* and *Sutterella* and enrichment of *Alistipes*, *Bacteroides*, *Clostridium_XI*, *Erysipelotrichaceae*, and *Escherichia* (Wang et al., 2015). A major limitation of these studies is the inclusion of mixed survivor cohorts, limiting our understanding of how the microbiome is impacted by (1) cancer type and (2) treatment type. Performing cohort studies and microbiome analyses in specialized survivor cohorts by controlling cancer type, treatment type, or both is required to better understand the impacts of these factors individually on gut microbiome composition and function. Furthermore, treatment duration may impact gut microbiome profiles and the regenerative capacity of the microbiome after treatment-induced injury. More studies are required to provide a more comprehensive understanding of short- and long-term gut microbiome compositional and functional changes in the context of cancer treatments and their link with GI health outcomes.

There is also an intricate relationship between the gut microbiome and mucosal immune system and. Gut microbiome signatures are shaped by immunotherapies and can also impact the efficacy of immunotherapy. For example, antibiotic-induced gut dysbiosis was linked with lower efficacy of ICIs in patients with lung cancer (Elkrief et al., 2024). Specific taxa, such as *Bifidobacterium* species and *Fb. prausnitzii*, have been associated with favorable health outcomes following immunotherapy, such as increased numbers of effector CD4^+^ (helper) and CD8^+^ (cytotoxic) T cells (Zakharevich et al., 2024). Other microbes like *Eubacterium rectale* are important for shaping responses to CTLA-4 blockade or anti-PD-1 therapy. The efficacy of CAR-T therapy has been linked with microbiome signatures in patients with cancer, reporting *Bifidobacterium*, *Prevotella*, *Sutterella*, *Ruminococcus*, *Akkermansia*, and *Collinsella* as key microbes driving CAR-T responses (Hu et al., 2022; Smith et al., 2022; Stein-Thoeringer et al., 2023). However, the gut microbiome-immune axis is bidirectional and the number of studies describing how immunotherapy may modulate the microbiome is extremely limited. Further mechanistic studies, such as fecal microbiota transplantation (FMT), are required to better understand the relationship between the gut microbiome composition and immunotherapy.

So far, we have reported that cancer survivors often experience CI and other brain dysfunctions, as well as poor GI health, including nausea, vomiting, and diarrhea and these changes are linked with the gut microbiome and mucosal immune system. Many of these studies are limited by a cross-sectional design and we cannot exclude the possibility of reverse causality. Although we suggest that changes in the microbiome may contribute to gut-brain impairments in cancer survivors, it is possible that these impairments also contribute to gut microbiome ecology. Additional studies are required to determine directionality between gut microbiome changes and gut-brain impairments in cancer survivors. Furthermore, we do not fully understand the mechanisms underlying cancer treatments and how they impact the gut and brain in survivors. Emerging research suggests that modulation of the gut-brain axis is central to both gut and brain health, including in cancer survivors who have undergone treatment.

## 4 Cancer treatments may impact gut and brain health via modulation of the gut-brain axis

The gut-brain axis is comprised of the bidirectional communications and signaling pathways connecting the gut and brain. Changes in the gut can impact the brain and vice versa. The gut-brain axis is comprised of physical connections/nerves (i.e., the vagus nerve) as well as biochemical routes including hormones (endocrine pathway), immune cells (immune pathway), neurotransmitters/neurons (neurological pathway), and metabolites (humoral/metabolic pathway) (Cryan et al., 2019). These connections between the gut and brain facilitate multiple aspects of physiology, including appetite and food intake, behavior, mood, and stress responses (Cryan et al., 2019; Appleton, 2018; Miller et al., 2023). Multiple studies have reported how the gut microbiome impacts the efficacy of cancer treatments; however, this is largely outside of the scope of this manuscript and has been reviewed elsewhere (Cheng et al., 2020). There are less studies investigating the impacts of cancer treatments on the microbiome, but it is suggested that microbiome modulation by cancer treatments plays a key role in the development of CI and chemobrain as well as gut dysfunctions in patients with cancer and survivors.

Although it is becoming more widely accepted that the gut microbiota is the central constituent of the gut-brain axis, it is less understood *how* the microbiota impacts gut and brain functions. The role of the gut microbiome in brain and cognitive functions has been long reported using proof-of-concept studies. Mice lacking gut microbiota (germ-free) exhibit impaired central and enteric nervous system development and stunted neurogenesis (Scott et al., 2020), dysregulated neurotransmitter turnover (Clarke et al., 2013; Heijtz et al., 2011), and altered sensory and motor functions, including in the gut (Husebye et al., 2001). Furthermore, microbiota disruption in early life and development is linked with changes in myelination and microglial morphology throughout life, indicating direct connections between gut microbial ecology and the brain (Lynch et al., 2023). Increases in bacteria such as Proteobacteria, *Enterobacteriaceae*, *Erysipelotrichaceae*, and *Akkermansia* and virus families including *Podoviridae* and *Siphoviridae* along with reductions in *Bifidobacterium* can also differentiate individuals with CI and/or AD from healthy controls (Chaudhari et al., 2023; Nagpal et al., 2019; Fan et al., 2023; Vogt et al., 2017; Nagpal et al., 2020; Li et al., 2019). In AD subjects with mild cognitive impairment (MCI), abundances of Proteobacteria were positively associated with Aβ42, a major neurotoxic peptide aggregate in AD (Nagpal et al., 2019). We recently reported that specific gut microbiome signatures, such as *Tyzzerella*, *Eggerthella lenta*, and *Bacteroides vulgatus* could differentiate cancer survivorship in older adults and were linked with cognitive function (Miller et al., 2025). Microbes like *Streptococcus* (*St.*) *thermophilus* and Firmicutes species were correlated with depleted neurotransmitter biosynthesis and energy metabolism, which could mechanistically contribute to CI (Miller et al., 2025). These studies suggest that the gut microbiome has a central role in the development of CI or neurodegenerative pathologies via modulation of the gut-brain axis. It is important to consider that most of these studies are observational, and the findings report only associations between gut microbiome signatures and cognitive or psychiatric health. Further work is warranted to establish causality and uncover the relationship between cancer treatments and the gut microbiome composition and function.

**Figure 1:**
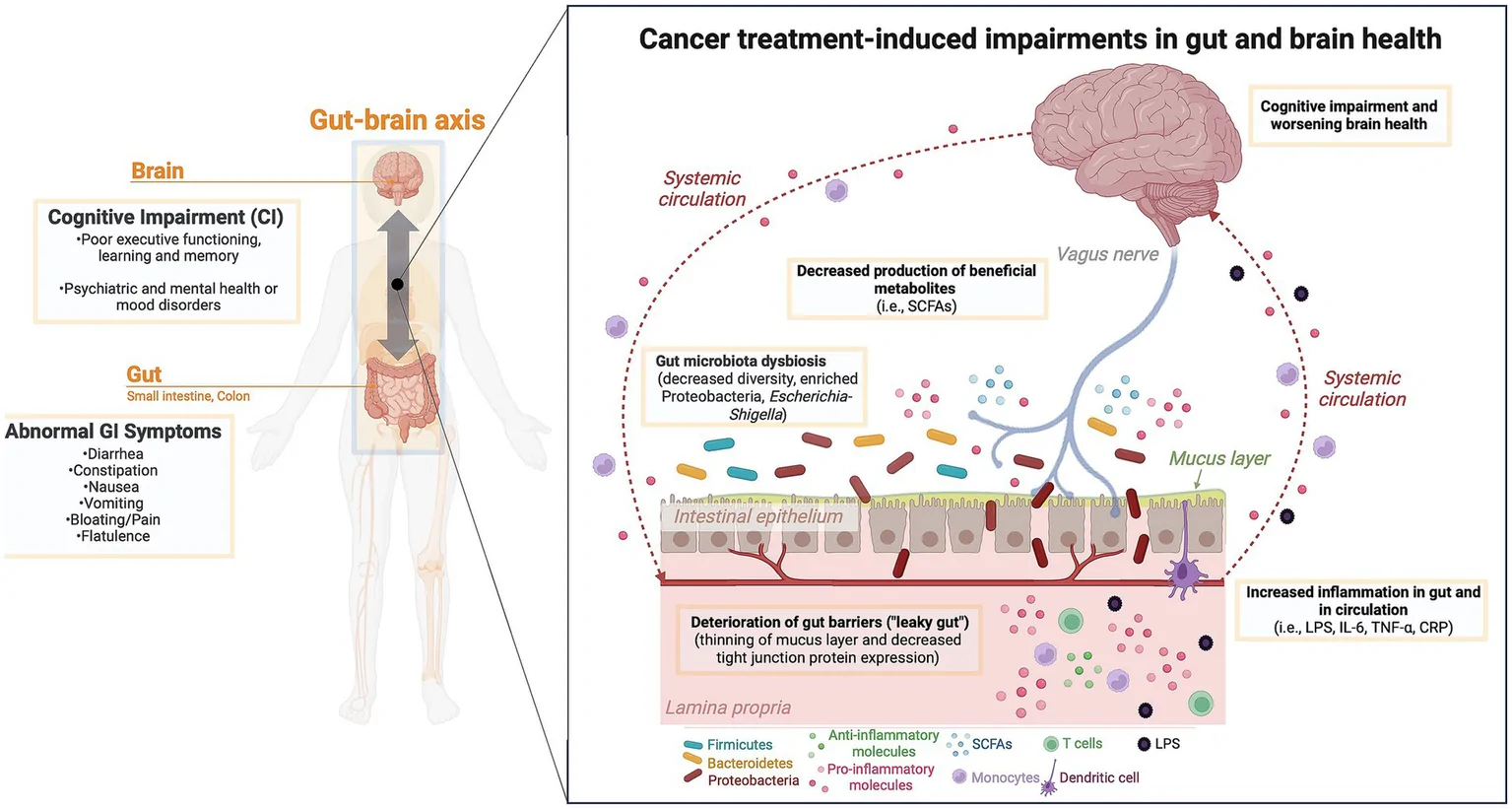
The physiological impacts of cancer treatments and their mechanisms in cancer survivors. Cancer treatments like chemotherapy, radiation, and immunotherapy can impart lingering and long-term effects on gut and brain health in cancer survivors, which include CI and bowel dysfunction (i.e., diarrhea, nausea, and bloating). Emerging evidence suggests that cancer treatments may induce these effects through modulation of the gut-brain axis. In particular, cancer treatments may induce gut microbiota dysbiosis, which lowers the production of beneficial metabolites produced by the gut microbiota, and leads to elevated gut permeability (“leaky gut”) and increased inflammation in the gut. Collectively, these changes in the gut may influence the brain and cognitive functions through systemic circulation or the vagus nerve. CI: Cognitive impairment, CRP: C-reactive protein, GI: Gastrointestinal, IL-6: Interleukin-6, LPS: Lipopolysaccharide, SCFAs: Short-chain fatty acids, TNF-*α*: Tumor necrosis factor-α. Figure adapted from (Miller, 2026).

The gut microbiota can affect the gut-brain axis by (1) modulating the immune system and inflammation, (2) regulating cellular stress (i.e., oxidative stress), (3) modulating gut or brain barrier integrity, (4) producing bioactive molecules or metabolites (i.e., SCFAs or secondary bile acids), or (5) synthesizing essential neurotransmitters including dopamine, serotonin, and γ-amino butyric acid (Figure 1) (Plaza-Diaz et al., 2019; Miller et al., 2024). These mechanisms of gut-brain axis modulation via the gut microbiome have been reported extensively elsewhere (Cryan et al., 2019). Herein, we will focus on gut microbiota-mediated immune system modulation and metabolite production, as these are the major mechanisms which are reported in interventional studies in cancer survivors.

### 4.1 Immune system modulation by the gut microbiota impacts brain health

The gut microbiota are in close proximity to immune cells lining the intestine, creating a dynamic interplay between these two systems. Immune cells maintain homeostasis in the gut via several mechanisms, such as production of antimicrobial peptides and regulation of toll-like receptor signaling, while the gut microbiota shapes immune system functions, including cytokine production and memory cell differentiation (Wu and Wu, 2012). Similar to how the microbiome changes as we age, the innate and adaptive immune systems change as we age, contributing to inflammaging, which is a hallmark of aging-related diseases (Prajapati et al., 2025a; Nagpal et al., 2018a). Another hallmark of these conditions is “leaky gut,” which connects the microbiome-immune axis with peripheral organs, including the brain, and impacts their physiological functions. When gut barriers are compromised, cellular components, including lipopolysaccharide (LPS, a major component of the Gram-negative bacterial cell wall), translocate from the intestine to systemic circulation, leading to peripheral inflammation or endotoxemia (Anhê et al., 2021; Mishra et al., 2024; Ahmadi et al., 2020). Immune cells or cytokines can pass through the blood–brain barrier, contributing to neuronal damage, neuroinflammation, and neurodegenerative disease progression (Prajapati et al., 2025a).

Several studies, including from our lab, have linked gut microbiome signatures with inflammation in CI and/or AD or other neurodegenerative diseases. Reduced microbial diversity, enrichment of *Dorea* and *Escherichia/Shigella*, and increased LPS have been linked with elevated serum inflammatory markers including IL-1β, CXCL2 (a chemokine), and IFN-γ as well as microglia activation or inflammasome formation in individuals with CI or brain amyloidosis (Liang et al., 2022; Cattaneo et al., 2017; Kitazawa et al., 2005; Minter et al., 2016). Brain IL-6 and TNF-α were positively correlated with *Helicobacter*, *Akkermansia*, and Verrucomicrobia abundances in an AD mouse model, further highlighting the potential of the microbiome-immune axis in brain functions (Prajapati et al., 2025b). Moreover, previous reports indicate that changing the gut microbiome via FMT or probiotics may improve CI or Parkinson’s disease pathology by attenuating neuroinflammation and blood brain barrier integrity via toll-like receptor and nuclear factor kappa B signaling, suppression of inflammatory cytokines like IL-6 and TNF-α, and reduction of serum LPS (Zhao et al., 2021; Yang et al., 2020). Similarly, salidroside attenuated CI in senescence-accelerated mouse prone 8 mice, which was linked with reductions in Aβ deposition, microglia activation, and circulating IL-1β, IL-6, and TNF-α levels as well as improved gut barrier integrity (Xie et al., 2020). Their findings suggest that these effects were mediated by the gut microbiome, including changes in the Bacteroidetes: Firmicutes ratio and depletion of inflammatory-linked microbes like *Clostridiales* and *Streptococcaceae* (Xie et al., 2020). These studies have started to highlight the central role of the gut microbiome-immune axis in CI and other brain pathologies.

The microbiome-immune axis is also modulated by cancer treatments, which impacts brain function. In a mouse model of cancer-related fatigue, enrichment of *Lactobacillus*, *Candidatus arthromitus*, and unclassified clostridia and reductions in *Escherichia*/*Shigella*, *Burkerholderia-Caballeronia-Paraburkholderia* and *Streptococcus* was linked with reduced fatigue, improved gut barriers, and reduced inflammation both in the intestine and brain (Lv et al., 2022). Furthermore, specific metabolic pathways (amino acid biosynthesis and taurine and β-alanine metabolism), cytokine levels, and tight junction protein (i.e., zonulin-1 and occludin) expressions were significantly correlated with gut microbiota signatures, demonstrating a clear link between the gut microbiota and gut-brain axis in a model of cancer treatment-induced fatigue (Lv et al., 2022). In female mice, glial fibrillary acidic protein (a marker of neuroinflammation) was correlated with *Ruminoclostridium_5* (positive) and *Lachnospiraceae* (negative) (Loman et al., 2019). Inflammation in the brain was also linked with colonic inflammation and atrophy, further underpinning connections between the gut, brain, and immune system (Loman et al., 2019). The gut microbiome composition is important for mediating the effects of immunotherapy, but the impacts of these microbiome-immune interactions on cognitive function in the context of cancer survivorship remain significantly understudied.

### 4.2 Modulation of the gut-brain axis by microbial metabolites

The gut microbiota carries out many metabolic functions, leading to the production of bioactive metabolites, which have several neuromodulatory effects, including on the immune system. SCFAs are 2–6 carbons in length and are produced via fermentation of dietary fibers by the gut microbiota. Butyrate, propionate, and acetate are the three major SCFAs present in the gut and constitute 60–70% of the energy required for colonocytes, making their role as signaling molecules and in maintaining mucosal immunity and barrier integrity indispensable (Recharla et al., 2023). Their modulatory effects can be facilitated through multiple mechanisms, including (1) as ligands of membrane G-protein coupled receptors such as free-fatty acid receptor 2/3 (FFAR 2/3) (Tolhurst et al., 2012; Mishra et al., 2020) or hydrocarboxylic acid receptor 2 (HCAR2) (Silva et al., 2020), (2) entrance into the cell via monocarboxylate transporters (i.e., MCTs) or sodium-coupled MCTs (Moschen et al., 2012), or (3) by impacting gene expression via inhibition of histone deacetylases (HDACs) (Zou et al., 2021). Many of these receptors are present in intestinal L- or enteroendocrine cells as well as in neuronal or glial cells. Furthermore, HDAC inhibition can occur in the brain, constituting a major epigenetic mechanism contributing to AD (Mishra et al., 2020; Samuel et al., 2008; Won et al., 2013; Silva et al., 2020; Elizondo-Vega et al., 2016; Yang et al., 2017). In individuals with AD and CI, propionate and butyrate were negatively correlated with Aβ42 or amyloid uptake, while valerate and acetate were positively correlated (Nagpal et al., 2019; Marizzoni et al., 2020). Similarly, supplementation with propionate or butyrate is associated with reductions in Aβ deposition and improvements in associative learning and cognitive function (Fernando et al., 2020; Govindarajan et al., 2011; Filippone et al., 2020; Tu et al., 2025). Butyrate has also demonstrated anti-inflammatory effects, rescued impairments in brain-derived neurotrophic factor and synaptic plasticity, and improved cognitive function via HDAC inhibition (Ji et al., 2014; Kilgore et al., 2010; Saw et al., 2020). Other studies have reported decreased levels of SCFAs in patients with CI or AD, which have been linked with inflammation (Cui et al., 2020; Wu et al., 2021). SCFAs are common byproducts of beneficial microbes such as *Lactobacillus* and *Bifidobacterium*, suggesting that abundances of these microbes were also negatively correlated with Aβ. Acetate was shown to regulate the maturation and activation of microglia (Erny et al., 2021; Erny et al., 2015), suggesting that microbial-derived SCFAs can modulate brain functions. Furthermore, reductions in acetate, propionate, butyrate, or the bacteria which produce them (i.e., *Allobaculum*, *Bifidobacterium*, and *Lactobacillus*) have been linked with increased neuroinflammation and CI via FFAR2/3 signaling (Mishra et al., 2024; Liao et al., 2022). Thus, there is a plausible relationship between gut microbial metabolism and cognitive function and/or brain neurochemistry – but these connections are significantly understudied, especially in humans. Mechanistic studies focusing on determining the causal relationships between microbiota, microbial-derived metabolites, and brain health are also warranted. Circulating SCFAs have been studied briefly in the context of cancer survivorship. In 59 patients with head and neck cancer, butyrate and isovalerate levels were negatively correlated with fatigue index and these were linked with changes in metabolic pathways related to inflammation and lipid and fatty acid biosynthesis (Xiao et al., 2023). More studies are required to confirm these findings and better understand the relationship between SCFAs and the gut microbiota-immune axis in cancer survivors.

There is a tight interplay between the gut microbiota, gut metabolome, and immunotherapy response. Microbial-derived butyrate is critical in driving the effects of cancer immunotherapies, including anti-PD-1 and immune checkpoint blockade, by altering T-cell receptor signaling and enhancing CD8^+^ T cell expansion and function in non-small cell lung cancer, glioma, and melanoma (Zhu et al., 2023; Li et al., 2025; Yang et al., 2025). Administering sodium butyrate has been linked with reduced M2 (inflammatory) macrophage polarization in synergy with PD-L1 blockade therapy – these immune system changes are also associated with reduced tumor burden and lower pathological tissue damage in CRC (Han et al., 2025). Single-cell transcriptomics and mouse studies revealed that the effects of butyrate were mediated through HDAC inhibition and modulation of toll-like receptor-4 signaling, thereby reducing polarization of M2 macrophages (Han et al., 2025). In another study on CRC, butyrate supplementation attenuated IFN-γ-mediated damage to CD8^+^ T cells, which enhanced their anti-tumor efficacy. Other SCFAs including acetate, propionate, and pentanoate have also demonstrated strong anti-tumor effects by mediating cancer immunotherapy response through T cell expansion and HDAC inhibition (Zhu et al., 2023; Luu et al., 2021). Bacterial-derived desaminotyrosine was linked with suppressed tumor growth during CTLA-4 treatment, with modulation of interferon signaling as a purported mechanism (Joachim et al., 2023). Furthermore, indole-3-carboxaldehyde, a tryptophan metabolite, was linked with improved gut barrier functions and reduced levels of inflammatory TNF-α, IL-1β, and IL-17A in ICI-induced colitis (Ciernikova et al., 2024). These studies highlight that SCFAs and other metabolites derived from the microbiota have strong roles in shaping immunotherapeutic responses to cancer, which may impact gut and brain health in cancer survivors via the gut-brain axis. However, more studies are warranted to improve our understanding of the impacts of immunotherapy on the gut microbiome. Most of the literature focuses on how baseline gut microbiota impact the efficacy of immunotherapy, but it is important to study microbiome-immunotherapy interactions bidirectionally and determine the longitudinal impacts of immunotherapy on gut microbial ecology post-treatment.

Collectively, these findings provide strong evidence of how the gut microbiome modulates the gut-brain axis to impact cognitive function and brain pathologies. They also highlight the gut-immune and gut-metabolome axes as cornerstone mechanisms contributing to gut-brain axis modulation. Therefore, gut microbiota modulation may be a feasible approach for improving gut and brain health in many diseases and may also improve health in cancer survivors. However, the number of studies is limited and sample sizes are small, restricting the generalizability of the findings. Many of these studies do not establish causality or directionality and so it is unclear if gut microbiota modulation impacts gut-brain pathologies or if impairments in gut-brain axis by cancer treatments shape the gut microbiota as well as the immune system and metabolome. Translational studies integrating gnotobiotic models, FMT, multi-omics analyses, and a longitudinal design are critical for better establishing causality.

## 5 Implementation of microbiome modulators in cancer survivors

**Figure 2:**
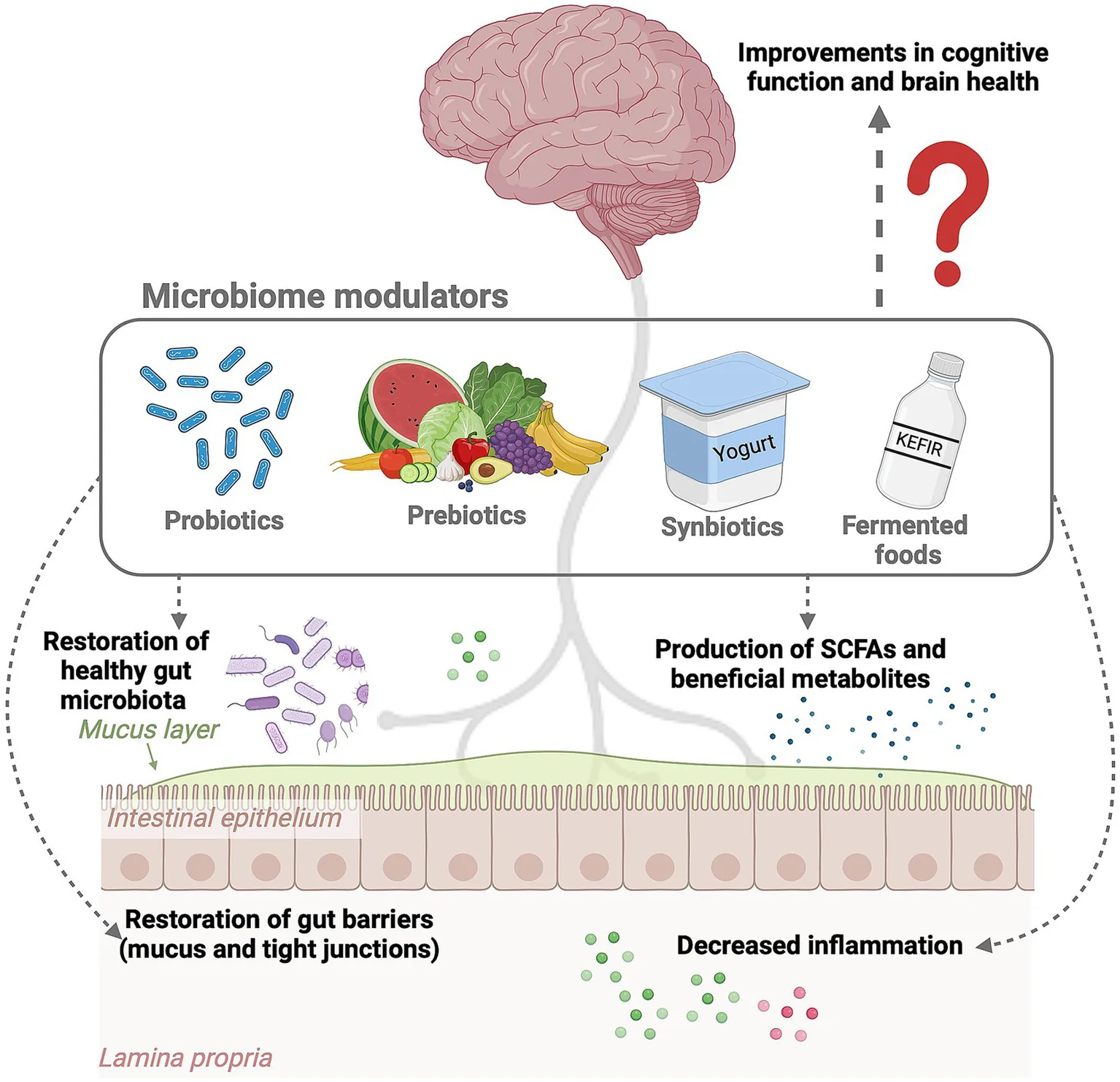
Microbiome modulators may improve gut and brain health through gut-brain axis modulation in cancer survivors. Microbiome modulators including probiotics, prebiotics, synbiotics, and fermented foods have the potential to improve CI and abnormal GI health induced by cancer treatments. Their mechanisms include restoration of healthy gut microbiota and beneficial metabolites, restoration and strengthening of gut barriers, and decreased inflammation. Further studies are warranted to elucidate if these microbiome modulators can improve cognitive functions and brain health in cancer survivors. CI: Cognitive impairment, GI: Gastrointestinal, SCFAs: Short-chain fatty acids.

Thus far, we have provided evidence that cancer survivors experience impairments in cognitive and GI health, which significantly lower QoL in many cancer survivors. However, there are no known long-term preventative or therapeutic agents which have been established to combat them. Microbiome modulators, including probiotics, prebiotics, synbiotics, and fermented foods (1) restore healthy gut microbiota (i.e., increase diversity and abundances of beneficial microbes like *Lactobacillus*) and strengthen gut barriers, (2) attenuate local and systemic inflammation, and (3) produce beneficial metabolites, which may improve QoL in cancer survivors (Figure 2). These microbiome modulators have been studied as therapeutic or preventative strategies for multiple conditions, including obesity (Nagpal et al., 2018b; Ahmadi et al., 2019; Yadav et al., 2013), diabetes (Miller et al., 2021; Yadav et al., 2007; Sabico et al., 2019), neurodegenerative diseases (Ahmadi et al., 2020; Prajapati et al., 2025b), psychosocial conditions (i.e., depression) (Ng et al., 2018), aging-related inflammation and CI (Yang et al., 2020; Ahmadi et al., 2020; Ruiz-Gonzalez et al., 2022), and other bowel-related diseases, in both animals and humans (Kim et al., 2019; Feng et al., 2023). They may demonstrate immunomodulatory effects by expanding microbial-derived metabolites like butyrate, improving the efficacy of immunotherapy treatment, reducing the burden of treatment-related gut-brain side effects, and improving QoL following treatment. However, their potential in cancer survivors remains largely unknown and the available literature is sparse. Microbiome modulators have been studied in survivors of multiple types of cancer, including breast, colorectal, head and neck, and prostate and bladder cancers, and we review the findings below (Table 2). The currently available studies provide preliminary evidence of the impacts of microbiome modulators in survivors of multiple types of cancer, but are limited by (1) small sample sizes, (2) heterogeneity in cancer type, treatment regimen, time-since-treatment, and other confounding variables, and (3) lack of long-term follow-up.

**Table 2:** Interventional studies reporting the use of microbiome modulators and their impacts on gut-brain functions and/or quality of life in cancer survivors.

Author and year	Study cohort and design	Intervention description, dose, and duration	Major findings with intervention
Probiotics			
Ohigashi et al. (2011)	Cohort: 63 survivors of colon (*n* = 40) or rectal (*n* = 23) cancer Cancer treatment: Surgery Study design: Single group, pre-post trial	Intervention: “The Guard”; *Lb. acidophilus* + *Ba. natto* Intervention start time: 6 months post-surgery Dose: 9 tablets/day: 30 mg/day *Lb. acidophilus* and 10 mg/day *Ba. natto* Duration: 3 months	63.5% reported significant bowel improvements Improved defecation frequency, diarrhea, constipation, and QoL
Lee et al. (2014)	Cohort: 60 survivors of CRC Placebo group (*n* = 32) Probiotic group (*n* = 28) Cancer treatments: Chemotherapy (*n* = 51) or chemotherapy + radiation (*n* = 9) Study design: Double-blind, randomized, placebo-controlled trial	Intervention: “Lacidofil”; *Lb. rhamnosus* R0011 + *Lb. acidophilus* R0052 Intervention start time: ~9-10 months post-treatment Dose: 1 tablet/day or 2 x 10^9^ CFU/day Duration: 12 weeks	Improved IBS symptoms, CRC-related FACT, fatigue, functional well-being and PHQ-9
Pellegrini et al. (2020)	Cohort: 34 breast cancer survivors MD only (*n* = 18, control group) MD + probiotics (*n* = 16) Cancer treatments: Not specified Study design: Randomized, open-label pilot intervention trial	Intervention: Probiotic sachet including *Bf. longum* BB536 and *Lb. rhamnosus* HN001 Intervention start time: Not specified Dose: 1 sachet/day Duration: 2 months	↑ total bacteria species and diversity ↓ Bacteroidetes / Firmicutes ratio Improved anthropometric and metabolic measures
Prebiotics			
Wierdsma et al. (2009)	Cohort: 16 head and neck cancer survivors Tube feeding (control, *n* = 10) Prebiotic group (*n* = 6) Cancer treatments: Chemotherapy and/or radiation Study design: Randomized, double-blind interventional trial	Intervention: FOS- and fiber-enriched tube feeding Intervention start time: Not specified Dose: *Ad libitum* Duration: 6 weeks	Stabilized GIQLI and fecal bifidobacteria Bifidobacteria levels correlated with GIQLI
Sheflin et al. (2017)	Cohort: 29 CRC survivors Control (*n* = 10) Rice bran (*n* = 9) Navy bean powder (*n* = 10) Cancer treatments: Chemotherapy and/or radiation Study design: Randomized-controlled pilot interventional trial	Intervention: 1. Heat-stabilized rice bran or 2. Cooked navy bean powder Intervention start time: > 4 months post-treatment Dose: 30 g/day (rice bran) or 35 g/day (bean powder) Duration: 4 weeks	↑ microbiome diversity and richness ↑ stool SCFAs and metabolic pathways related to fatty acids and monosaccharides
Zhang et al. (2023)	Cohort: 48 survivors of colon, rectal, or colorectal cancers Intervention first (*n* = 28) Control first (*n* = 20) Cancer treatments: Not specified Study design: Randomized crossover and open-label trial	Intervention: Pressure-cooked and canned organic navy beans Intervention start time: Not specified Dose: 1 cup/day of navy beans (16 g dietary fiber, 14 g protein, 220 kcal per serving) Duration: 8 weeks, either preceding or following 8 weeks of no intervention (crossover design)	↑ microbiome diversity and abundances of *Faecalibacterium*, *Eubacterium*, and *Bifidobacterium* ↑ pipecolic acid, FGF-19 ↓ indoles, IL-10Rα Favorable microbiome and metabolome shifts reversed after ending intervention
Cares et al. (2025)	Cohort: 13 survivors of leukemia, lymphoma, or sarcoma TRE (*n* = 6) Prebiotics + TRE (PreTRE, *n* = 7) Cancer treatments: Not specified Study design: Randomized-controlled pilot interventional trial	Intervention: Prebiotic sachet containing Bimuno^®^ and active GOS Intervention start time: > 4 months post-treatment Dose: 3.65 g daily Bimuno^®^ and 2.75 g active GOS Duration: 12 weeks	↓ constipation, diarrhea, and gas and bloating symptoms in PreTRE from baseline to follow-up ↓ cardiometabolic parameters (i.e., insulin, cholesterol, hs-CRP, HbA1c, and triglycerides) in PreTRE after 12 weeks
Synbiotics			
Vafa et al. (2020a)	Cohort: 121 breast cancer survivors with lymphedema Control (*n*o intervention, *n* = 41) CR + placebo (*n* = 39) CR + synbiotic (*n* = 41) Cancer treatments: Chemotherapy, radiation, surgery, or hormone therapy Study design: Parallel, randomized, placebo-controlled intervention trial	Intervention: Synbiotic capsules containing *Lb. casei*, *Lb. acidophilus*, *Lb. bulgaricus*, *Lb. rhamnosus*, *Bf. breve*, *Bf. longum*, and *St. thermophiles* and FOS or placebo (lactose) Intervention start time: ≥ 6 months post-treatment Dose: 10^9^ CFU/day and 38.5 mg FOS Duration: 10 weeks	Improved QoL scores, including psychosocial and functional domains (synbiotic group) ↓ edema volume and BMI in synbiotic group compared to control
Vafa et al. (2020b)	Cohort: 80 breast cancer survivors with unilateral arm lymphedema Placebo + LCD (*n* = 39) Synbiotic + LCD (*n* = 41) Cancer treatments: Surgery and/or chemotherapy and/or radiation Study design: Randomized double-blind controlled interventional trial	Intervention: Synbiotic capsules containing *Lb. casei*, *Lb. acidophilus*, *Lb. bulgaricus*, *Lb. rhamnosus*, *Bf. breve*, *Bf. longum*, and *St. thermophiles* and FOS Intervention start time: ≥ one year post-treatment Dose: 10^9^ CFU/day and 38.5 mg FOS Duration: 10 weeks	↓ edema volume after synbiotic compared to baseline ↓ hs-CRP, IL-1β, and leptin after synbiotic compared to baseline ↓ TNF-α and leptin in synbiotic compared to placebo
Raji Lahiji et al. (2021)	Cohort: 72 overweight/obese postmenopausal breast cancer survivors Placebo (*n* = 36) Synbiotic (*n* = 36) Cancer treatments: Chemotherapy and/or radiation Study design: Randomized, triple-blind, placebo-controlled interventional trial	Intervention: Synbiotic containing *Lb. casei*, *Lb. acidophilus*, *Lb. rhamnosus*, *Lb. bulgaricus*, *Bf. breve*, *Bf. longum*, *St. thermophilus* and FOS Intervention start time: ≥ one month post-treatment Dose: 10^9^ CFU/day and 35 mg FOS Duration: 8 weeks	↑ serum adiponectin (synbiotic) ↓ serum TNF-α and hs-CRP levels (synbiotic)
Saneei Totmaj et al. (2022)	Cohort: 88 obese or overweight breast cancer survivors with lymphedema Placebo + LCD (*n* = 44) Synbiotic + LCD (*n* = 44) Cancer treatments: Chemotherapy and/or radiation Study design: Randomized, double-blind, placebo-controlled interventional trial	Intervention: Synbiotic containing *Lb. casei*, *Lb. acidophilus*, *Lb. rhamnosus*, *Lb. bulgaricus*, *Bf. breve*, *Bf. longum*, and *St. thermophilus* and FOS Intervention start time: ≥ six months post-treatment Dose: 10^9^ CFU/day and 38.5 mg FOS Duration: 10 weeks	↓ edema volume (synbiotic) ↓ body weight, BMI, body fat percentage, and waist circumference (both groups) ↓ VEGF (synbiotic) ↑ adiponectin and IL-10 (synbiotic)
Fermented foods			
Henriksson et al. (1995)	Cohort: 39 survivors of prostate and/or bladder cancer with chronic bowel discomfort Placebo group (*n* = 19) Fermented milks group (*n* = 20) Cancer treatments: Irradiation Study design: Randomized, double-blind interventional trial	Interventions: Fermented milks – 1. Verum halsofil (contains *Lc. lactis* and *Lc. cremoris*) or 2. Norrlandsfil (contains *Lc. lactis*, *Lc. diacetylactis*, *Leuconostock*, and *Lc. cremoris*) Intervention start time: ≥ one year post-treatment Dose: 300 mL, twice per day Duration: 5 weeks	↓ number of stools/day GI discomfort improved in >50% with fermented milk
Smoak et al. (2021)	Cohort: 24 survivors of breast, prostate, endometrial, colon, gastroesophageal, tonsil, renal, ovarian, or blood cancer: Control group (*n* = 12) Kefir group (*n* = 12) Cancer treatments: Chemotherapy (varying), radiation (external beam), or combination of both Study design: Non-randomized, controlled intervention trial	Intervention: Kefir containing *Lb. lactis*, *Lb. rhamnosus*, *St. diacetylactis*, *Lb. plantarum*, *Lb. casei*, *Sc. florentinus*, *Lc. cremoris*, *Bf. longum*, *Bf. breve*, *Lb. acidophilus*, *Bf. lactis*, and *Lb. reuteri* Intervention start time: Within 2 years of treatment Dose: 8 oz. following each exercise session, approximately 3 times/week (15-20 billion CFU/8 oz.) Duration: 12 weeks	Improved lean body mass ↓ depression, fatigue, and GI distress ↓ circulating LPS and non-classical monocytes ↑ classical monocytes

Ba.: Bacillus, Bf.: Bifidobacterium, BMI: Body mass index, CFU: Colony-forming unit, CR: Calorie restriction, CRC: Colorectal cancer, FACT: Functional Assessment of Cancer Therapy, FGF-19: Fibroblast growth factor-19, FOS: Fructooligosaccharides, GI: Gastrointestinal, GIQLI: Gastrointestinal Quality of Life Index, GOS: Galactooligosaccharides, HbA1c: Hemoglobin A1c, hs-CRP: High-sensitivity C-reactive protein, IBS: Irritable bowel syndrome, IL-1β: Interleukin-1β, IL-10Rα: Interleukin-10 receptor-α, Lb.: Lactobacillus, Lc.: Lactococcus, LCD: Low calorie diet, LPS: Lipopolysaccharide, MD: Mediterranean diet, PHQ-9: Patient Health Questionnaire-9, QoL: Quality of life, Sc.: Saccharomyces, SCFA: Short-chain fatty acid, St.: Streptococcus, TNF-α: Tumor necrosis factor-α, TRE: Time restricted eating.

### 5.1 Probiotics

Probiotics are live microorganisms which may confer beneficial effects in humans when consumed adequately (Hill et al., 2014). Their effects are strain-specific and probiotics must be (1) safe and non-pathogenic, (2) able to survive in acidic environments including the gut, and (3) have resistance against bile salts and enzymes (Nagpal et al., 2012; Colombo et al., 2018). Several probiotics are anti-pathogenic (i.e., against pathogens like *Helicobacter pylori*) and resistant against common antibiotics, but these features are not compulsory (Nagpal et al., 2012). Probiotics may improve health by colonizing the gut and attaching to mucus (Miller et al., 2024), restoring the abundances of health-promoting bacteria (i.e., *Lactobacillus* and *Bifidobacterium*), and producing beneficial metabolites like SCFAs (Yadav et al., 2013; Yadav et al., 2022). Probiotics can also affect brain functions via the gut-brain axis (Cryan et al., 2019). We reported that a 10-strain probiotic cocktail containing lactobacilli and enterococci significantly improved gut dysbiosis and protected from aging-related inflammation and leaky gut through SCFA production in mice (Nagpal et al., 2018b; Ahmadi et al., 2020). Our multi-strain and novel probiotics have also protected from AD-like pathologies, including Aβ deposition, neuroinflammation, and CI (Prajapati et al., 2025b). Emerging human studies have also investigated the potential of probiotics on metabolic and neurological health, highlighting the necessity for such studies in cancer survivors (Zikou et al., 2023; Lee et al., 2022).

The potential of probiotics has been investigated in survivors of breast cancer and CRC. Two-month supplementation of *Bifidobacterium* (*Bf.*) *longum BB536* and *Lactobacillus* (*Lb.*) *rhamnosus HN001* with Mediterranean diet in overweight breast cancer survivors was linked with significant increases in total bacteria counts and microbiome diversity with concurrent decreases in the Firmicutes to Bacteroidetes ratio (Pellegrini et al., 2020). Furthermore, *Eubacterium* and *L-Ruminococcus* (belongs to family *Lachnospiraceae*) were significantly enriched in the gut of cancer survivors taking probiotics, while *Bacteroides* and *Butyiricoccus* were depleted. Serum C-reactive protein (CRP) levels were significantly reduced in the probiotic-fed group, compared to controls, suggesting that *Bf. longum* and *Lb. rhamnosus* may have immunomodulatory effects (Pellegrini et al., 2020). Elevated CRP is associated with low-grade, consistent inflammation, which is a hallmark of cancer pathology (Hart et al., 2020). In CRC survivors who were up to 2 years post treatment, 12-week supplementation of *Lb. rhamnosus* and *Lb. acidophilus* together was associated with significant reductions in irritable bowel symptoms (45.7% vs. 62.5% in the probiotic and control groups, respectively) and improvements in QoL, according to the FACT and PHQ-9 (Lee et al., 2014). Colon or rectal cancer survivors who had undergone surgery experienced significant improvements in stool frequency/form, reduced abdominal pain, and higher QoL following 3-month intervention with *Bacillus natto and Lb. acidophilus* probiotics (Ohigashi et al., 2011). Despite these emerging findings, none of these studies investigated changes in the gut microbiome composition due to probiotics or explored their mechanisms. An ongoing randomized, single-blinded, placebo-controlled trial in Australia (ClinicalTrials ID: NCT07363057) is investigating the impacts of a *Lactobacillus*- and *Bifidobacterium*-based probiotic for improving immune cell subsets, physical function, gut microbiome composition, and cognitive health in adult cancer survivors. Such studies will provide better insight on the connections between the gut microbiome, immune system, and cognitive health in cancer survivors. Nonetheless, additional trials are necessary to study impacts of these strains on the microbiome as well as explore mechanisms by integrating models like *Caenorhabditis elegans* (*C. elegans*) or mice with human data. Future studies also should investigate the potential of probiotics on cognitive or psychosocial health in cancer survivor cohorts.

### 5.2 Prebiotics

Prebiotics are water soluble and non-digestible dietary fibers, such as fructo-oligosaccharides and inulin. They are fermented by the gut microbiota to produce SCFAs and other bioactive metabolites and are a major energy source for colonocytes and the microbiota (Hill et al., 2014; Den Besten et al., 2013). Prebiotics must be (1) resistant to digestive enzymes, acids, and intestinal absorption, (2) able to be fermented by the gut microbiota, and (3) able to stimulate the growth of beneficial gut microbes (Hill et al., 2014; De Vrese and Schrezenmeir, 2008). We reported that prebiotics isolated from acorn and sago improved insulin resistance and inflammation via SCFA production (Ahmadi et al., 2019). Several other studies have reported that prebiotics modulated metabolic and immune system pathways via their antioxidant, anti-diabetic, and anti-obesogenic properties (Xu et al., 2015; Chaudhary et al., 2016; Beylot, 2005; Canfora et al., 2017; Genta et al., 2009; Ahmadi et al., 2017). However, the effects of prebiotics in the context of gut-brain health in cancer survivors remain significantly understudied.

Supplementation with fructo-oligosaccharides (FOS) and fiber during tube feeding in head and neck cancer survivors (*n* = 6) who had undergone radiation or surgery was linked with stable GI-specific QoL scores and fecal bifidobacteria levels compared to controls (Wierdsma et al., 2009). Bifidobacteria are common probiotics with reported benefits in CRC, diarrhea, and inflammatory bowel disease, largely through SCFA production (O'callaghan and Van Sinderen, 2016). Therefore, prebiotic supplementation may have stabilized gut health by maintaining *Bifidobacterium* abundance. However, larger cohort studies are warranted to confirm these findings. Twelve-week consumption of galacto-oligosaccharide (GOS)-based prebiotics was associated with decreased occurrence of GI symptoms including constipation, diarrhea, and bloating, and improved cardiometabolic parameters like fasting insulin, cholesterol, and high sensitivity-CRP (hs-CRP) (Cares et al., 2025). Additionally, in CRC survivors who had previously undergone chemotherapy and/or radiation, consumption of navy bean powder was linked with increased microbial species richness (Sheflin et al., 2017). Consumption of heat-sterilized rice bran was associated with significant enrichment of Bacteroidetes, depletion of Firmicutes and a lower Firmicutes: Bacteroidetes ratio, which were all linked with changes in microbial metabolism and increased acetate (Sheflin et al., 2017). In a recent crossover trial, intervention with organic navy beans was linked with increased microbiome diversity and enrichment of *Faecalibacterium*, *Eubacterium*, and *Bifidobacterium*, which are potential SCFA-producing bacteria (Zhang et al., 2023). Increased levels of pipecolic acid and fibroblast growth factor-19 and decreased abundances of indole derivatives were correlated with these microbiome signatures in CRC survivors (Zhang et al., 2023). These findings suggest that navy beans or rice bran may exert benefits on CRC survivors via gut microbiome modulation and changes to microbial metabolism, specifically through SCFA production. However, larger randomized controlled trials in other specialized cohorts are necessary to confirm these findings and better elucidate the potential benefits of prebiotics in cancer survivors.

### 5.3 Synbiotics

Synbiotics are the synergistic combination of probiotics and prebiotics which confer health benefits on the host (Swanson et al., 2020). A synbiotic containing *Lb. acidophilus*, *Bf. lactis*, *Bf. longum*, and *Bf. bifidum* and GOS increased microbiota diversity, enriched *Lactobacillus* and *Bifidobacterium*, and decreased blood glucose levels in obese individuals (Sergeev et al., 2020). We reported that a synbiotic yogurt containing five human-origin lactobacilli probiotics and prebiotics isolated from sago significantly protected from high-fat diet- and streptozotocin-induced diabetes in mice via gut microbiome modulation (Miller et al., 2021). Synbiotics often have stronger antioxidant and anti-inflammatory effects compared to pro- or prebiotics alone through their synergistic effects (Sharma et al., 2019). Because prebiotics serve as substrates for probiotics, synbiotics may exert their beneficial effects by enriching the abundance of beneficial bacteria, contributing to SCFA or bioactive metabolite production.

In overweight/obese breast cancer survivors, 8-week supplementation of a synbiotic containing *Lb. casei, Lb. acidophilus, Lb. rhamnosus, Lb. bulgaricus, Bf. breve, Bf. longum*, and *St. thermophilus* probiotics and FOS was associated with a significant increase in serum adiponectin, with concurrent reductions in TNF-α and hs-CRP levels (Raji Lahiji et al., 2021). Adiponectin is an anti-inflammatory, antioxidative, and anti-fibrotic adipokine (produced by adipocytes) which regulates glucose levels, lipid metabolism, and insulin sensitivity (Nguyen, 2020). On the other hand, TNF-α and hs-CRP are classical pro-inflammatory markers and are elevated in many conditions, including obesity, diabetes, and neurodegenerative diseases (Miller et al., 2021; Mishra et al., 2024). Therefore, these findings suggest that this synbiotic modulated the immune system; however, no outcome measures, including GI health, microbiome composition, cognitive function, or QoL were assessed. Ten-week supplementation of the same synbiotic in breast cancer survivors with lymphedema significantly decreased edema volume as well as levels of hs-CRP, IL-1β, and leptin (Saneei Totmaj et al., 2022; Vafa et al., 2020a). Compared to the placebo group, TNF-α and leptin levels were significantly lower in the synbiotic group after 10 weeks (Vafa et al., 2020a). There were no significant differences in adiponectin, IL-10, transforming growth factor-*β*, and vascular endothelial growth factor. In combination with calorie restrictive feeding, 10-week supplementation with synbiotics significantly improved QoL, including psychosocial and functional domains, as well as lowered edema volume and body mass index compared to controls (no intervention) (Vafa et al., 2020b). It cannot be concluded that the reported effects were due to the synbiotics alone or were in part a result of calorie restriction. Collectively, these studies indicate that synbiotics may have immunomodulatory and metabolic effects, but additional cohort studies with larger sample sizes and long-term follow-up are needed to confirm these findings. Future studies should also include outcome measures of gut and brain health- as well as microbiome composition- to determine if these synbiotics have significant neuromodulatory effects and impacts on the gut microbiome in cancer survivors.

### 5.4 Fermented foods

Fermented foods are produced through controlled microbial growth and include foods and drinks such as yogurt, kefir, kombucha, sauerkraut, and kimchi (Dimidi et al., 2019). Most fermented foods contain probiotic strains, such as *Lactobacillus* or *Saccharomyces* (yeast), which may exert health benefits by outcompeting pathogens or producing neuro- or immunomodulatory byproducts or metabolites like SCFAs during fermentation (Leeuwendaal et al., 2022). Previous studies report that fermented foods can (1) combat intestinal infections caused by pathogens like *Helicobacter pylori* and *Campylobacter jejuni* (Bekar et al., 2011; Sreeramulu et al., 2000), (2) attenuate diarrhea and constipation (Merenstein et al., 2009), and (3) improve inflammatory bowel disease symptoms (Yılmaz et al., 2018; Nielsen et al., 2018), hyperlipidemia, hyperglycemia (Aloulou et al., 2012; Korem et al., 2017; Miller et al., 2021), cancer pathology, and psychiatric conditions. However, in the context of cancer survivorship, the precise impacts and mechanisms of fermented foods remain largely unknown.

In a cohort of breast cancer survivors (*n* = 606), intake of fermented soy and/or dairy products was negatively correlated with cancer reoccurrence and mortality (Yang J. et al., 2023). Although these results suggest that supplementation of fermented foods may reduce the risk of mortality or cancer relapse, this study did not include any measures of gut or brain health. It is critical to determine how these supplements may impact GI, mental, or psychosocial health in cancer survivors. Consumption of fermented milk drinks (containing *Lactococcus* (*Lc.*) *lactis* and *Lc. cremoris* or *Lc. lactis*, *Lc. diacetylactis*, and *Leuconostoc* isolates) was linked with significant improvements in bowel discomfort in survivors of prostate and/or bladder cancers who had previously received radiation (Henriksson et al., 1995). Supplementation of kefir (containing *Lc. lactis*, *Lb. rhamnosus*, *St. diacetylactis*, *Lb. plantarum*, *Lb. casei*, *Saccharomyces florentinus*, *Leuconostoc cremoris*, *Bf. longum*, *Bf. breve*, *Lb. acidophilus*, *Bf. lactis*, and *Lb. reuteri*) plus exercise for 12 weeks was associated with increases in lean body mass, and reduced symptoms of depression, fatigue, and gastric distress in breast cancer survivors, compared to controls (Smoak et al., 2021). Furthermore, systemic LPS was significantly reduced in the kefir-exercise group compared to controls (Smoak et al., 2021). LPS is highly inflammatory, stimulates the production of cytokines like IL-6, IL-1β, and TNF-*α*, and has been reported in many inflammatory conditions, including in the gut and brain (Tucureanu et al., 2018; Anhê et al., 2021). This finding, along with increased monocyte levels, suggests that the kefir-exercise regimen modulated the immune system. However, this study is limited by its non-randomized design and it cannot be concluded that kefir alone produced these effects. An ongoing trial in Taiwan (ClinicalTrials ID: NCT05717972) is investigating the impacts of kombucha consumption on psychosocial health and sleep quality in breast cancer survivors. However, additional controlled trials with larger sample sizes and a randomized design are necessary to confirm these findings and establish the utilization of fermented foods for improving QoL, including gut-brain health, in cancer survivors. Evaluations of gut microbiome ecology are important for determining the impacts of fermented foods in cancer survivors and elucidating relationships between the gut microbiome, its metabolism, and the immune system.

## 6 Limitations and future directions

This manuscript has reviewed the impacts of cancer treatments on gut-brain health and QoL in cancer survivors through the microbiome-immune axis and the potential of microbiome modulators. Treatment-related side effects place significant physical, economic, and social burdens on cancer survivors, their families, and healthcare systems. Despite the compelling evidence presented herein, there are some limitations which should be addressed.

### 6.1 Limitations related to human cohort studies and sample sizes

CI and gut dysfunctions have been reported in survivors of multiple cancer types, including breast, colon/colorectal, testicular, gynecologic, prostate, and head and neck cancers. Interventional studies also suggest that microbiome modulators, such as probiotics, prebiotics, synbiotics, and fermented foods, may improve microbiome, gut, immune, and QoL outcomes in cancer survivors. However, the evidence in cancer survivor populations is sparse and available studies are limited by small sample sizes. Larger observational and interventional studies are required to gain a more comprehensive understanding of microbiome-gut-brain impairments in the cancer survivor population and the therapeutic potential of microbiome-based interventions.

### 6.2 Limitations regarding heterogeneity in cohorts, study design, and outcome measures

The studies presented herein are further limited by significant heterogeneity in cohort demographics, study design, and outcome measures. This includes variability in time since treatment, cancer treatment type, and intervention duration and follow-up.

#### 6.2.1 Confounding variables and heterogeneity across cohorts

Several factors, including age, gender, education level, lifestyle factors like smoking or drinking, prevalence of comorbidities, and genetics, have significant influence on the gut microbiome, immune functions, and gut and brain function, irrespective of cancer history. Some studies in this review have used regression analysis or propensity score matching to address these confounding variables and others, but additional studies with similar analyses are warranted. Furthermore, gut and brain functions or the impacts of microbiome modulators may vary across survivors of various cancer types (Jain, 2020). Additional studies involving specialized cohorts of cancer survivors of all types are necessary to delineate the effects of microbiome modulators based on cancer type. Cancer treatments are often complex due to cancer type, stage, and severity, and are highly personalized, which may impact the prevalence of CI and gut or brain dysfunctions as well as the efficacy of microbiome modulators and more studies are needed to determine the influence of cancer treatment type. These studies will aid in the long-term establishment and widespread utilization of precision-based microbiome modulators in cancer survivors.

#### 6.2.2 Heterogeneity in study design and outcomes

Many of the observational studies presented include a cross-sectional design, limiting our understanding of how gut microbiome, immune, and cognitive/psychosocial markers change over time post-treatment. Some studies include multiple assessments post-treatment, but there are significant gaps between measurements. Furthermore, there is variability in the follow-up times from study-to-study, making it challenging to establish a clear window for delivering post-treatment therapeutics. Future studies should include short- and long-term longitudinal follow-ups so we can gain a better understanding of the precise changes happening in microbiome, gut, brain, and immune functions following treatment.

Additionally, some studies assess microbiome as a primary outcome, while others focus on metabolites or inflammatory biomarkers, creating heterogeneity in outcome measures across cohorts. Although CI (or “chemobrain”) is commonly reported in cancer survivors – even for decades post-treatment – none of the interventional studies presented here have investigated the effects of microbiome modulators on cognitive health in cancer survivors. Therefore, future studies should include cognitive health outcomes using validated tools such as the MoCA, MMSE, or FACT-Cog as well as techniques like MRI or positron emission tomography. Conducting interventional studies in larger and more diverse cancer survivor cohorts and including consistent study designs and outcomes related to microbiome, gut, and brain health will provide stronger evidence of the potential of microbiome modulators in cancer survivors.

#### 6.2.3 Heterogeneity in time of intervention

This review focuses on gut-brain impairments and microbiome-based interventions in cancer survivors, which are defined as individuals who have already completed their treatment. The interventional studies included in this review began interventions between three months and two years following treatment. As reported in observational studies, cognitive, psychiatric, and gut health impairments may be short-term in some individuals, but persistent in others; thus, outcomes measured at 6 months post-treatment can be significantly different from those measured one or two years later. The lack of robust longitudinal studies limits the ability to establish an optimal intervention window for delivering microbiome-based regimens.

The timing of introducing microbiome-based interventions respective to cancer therapy has important clinical implications and should be considered. Interventions which begin immediately after treatment may target recovery and restoration by reducing persistent treatment-related symptoms, supporting immune and mucosal repair, improving dietary tolerance, and facilitating return to baseline health. On the other hand, interventions administered later in survivorship may be aimed at improving chronic health outcomes, including CI, which has been reported up to 20 years or more post-treatment in some cohorts. These interventions may be preventive, potentially reducing the risk of recurrence or the emergence of other treatment-related complications.

Determining the impacts of microbiome-based interventions during active cancer treatment has meaningful clinical implications, but this is outside the scope of this manuscript and has been reviewed elsewhere (Deleemans et al., 2021). Interventions delivered during active treatment may be designed as supportive care strategies aimed at preventing or reducing treatment-related toxicities, such as intestinal mucosal injury, immune dysregulation, and CI. However, there are significant safety considerations associated with delivering probiotics during active cancer treatment, such as interference with treatment efficacy, altered drug metabolism, and increased risk of immune activation, microbial translocation, or infection (i.e., bacteremia). These concerns may be especially relevant for those receiving immunotherapy because the gut microbiome may influence anti-tumor response as well as the development of immune-related adverse events, such as diarrhea or colitis (Spencer et al., 2021; Hwang et al., 2023). In contrast, delivering microbiome interventions during survivorship may be more favorable because immune-modulating therapy is no longer being administered and there is a lower risk of treatment-related interactions. Caution may still be warranted for survivors with persistent immunosuppression or other high-risk conditions.

### 6.3 Limitations regarding adverse events and probiotic safety

Among the studies presented in this manuscript, there were no adverse or life-threatening effects reported. Mild GI-related symptoms, such as excessive bloating or gassiness, were reported in some studies; although uncomfortable, these side effects are non-life threatening and do not impact the safety of -biotic regimens. Despite the general consensus that probiotics are safe, adverse effects may occur in immunocompromised individuals, including cancer survivors. Individuals with cancer or survivors often have weakened immune systems and are more susceptible to infections, which can be life-threatening, such as with sepsis or bacteremia (Przybylski and Reeves, 2017). The risk of adverse events must be considered when determining microbiome-based interventions, particularly those with probiotics, for cancer survivors.

The limited number of interventional studies also contributes to a lack of clear criteria for strain selection. Whether given as live bacteria or in a fermented food, the effects of probiotics are highly strain-specific (Nagpal et al., 2012). Microbiome-based interventions are highly personalized because their efficacy can vary across individuals due to many factors, including baseline microbiome composition, dietary patterns and lifestyle habits, disease history, cancer/treatment type, and time since treatment. Together, these factors make it difficult to establish clear strain selection criteria for cancer survivors. Conducting additional large-scale intervention studies with (1) a robust study design, (2) consistent outcome measures, including adverse events, and (3) a rationale for strain selection will aid in a more widespread establishment of these criteria. Preclinical studies also are imperative for comprehensively evaluating the safety and efficacy of microbiome-based interventions, particularly those containing probiotics, for individuals undergoing cancer treatments and/or survivors.

### 6.4 Limitations regarding causality and publication bias

Although current evidence suggests that the gut microbiome is impacted by cancer treatments to modulate gut and brain health, these studies do not establish causality. Most of the findings are based on associations between gut microbiome signatures and inflammatory, cognitive, and/or psychosocial outcomes. Furthermore, the potential of reverse causality cannot be excluded. The gut-brain axis is bidirectional and impairments in cognitive and psychosocial functions may also contribute to changes in the microbiome composition. Additional longitudinal studies with repeated microbiome and clinical assessments are required to determine directionality between gut microbiome changes and gut-brain impairments in cancer survivors.

Preclinical approaches, including FMT and gnotobiotic mouse models, may further strengthen our understanding of the causal role of the microbiome and its modulators in the context of cancer treatments and survivorship. FMT studies from human donors into mice and in clinical trials have demonstrated the strain-specific immunomodulatory roles of the gut microbiota in the context of immunotherapy efficacy (Tanoue et al., 2019; Davar et al., 2021). These studies support the potential of using FMT to better define causal relationships.

There is also the possibility of publication bias. Studies reporting significant microbiome differences, favorable effects of probiotics, prebiotics, synbiotics, fermented foods, or positive associations with gut, brain, or QoL outcomes may be more likely to be published than studies with negative or inconclusive findings. This is particularly relevant in an emerging field where many studies are small, exploratory, or pilot in nature. As a result, the available literature may overestimate the consistency and efficacy of microbiome-based interventions in cancer survivors. Future studies and reviews should integrate comprehensive literature searches (including “gray literature” such as conference abstracts and dissertations), reviews of clinical trial registries, and meta-analytic approaches such as funnel plots and Egger’s test.

### 6.5 Future scope of microbiome-based interventions in cancer survivorship

Even though some studies report the use of microbiome modulators, very few investigate changes in microbiome diversity and taxonomy following treatment. There are multiple technologies to profile the microbiome, including 16S rRNA sequencing and metagenomics, making it feasible to investigate mechanisms of cancer treatments on gut microbiota and how they contribute to gut and brain health. Both 16S and metagenomic pipelines allow us to assess compositional changes in the microbiome, but shotgun metagenomics also provides insight on microbial functional potential.

Postbiotics, which are defined as inactive and/or dead probiotics and their constituents or metabolites, may be a more feasible approach for individuals with weaker immune systems, including cancer survivors (Salminen et al., 2021). Because postbiotics are non-viable, they do not replicate and may reduce risks associated with live microbial interventions, such as microbial translocation, transfer of antibiotic resistance genes, or the development of enteric and/or bloodstream infections. Postbiotics derived from *Lactobacillus* and *Bifidobacterium* can ameliorate aging-related CI, leaky gut, and inflammation or metabolic conditions via microbiome modulation (Wang et al., 2020; Shin et al., 2010; Martorell et al., 2021; Balaguer et al., 2022; Kolling et al., 2018; Compare et al., 2017). Furthermore, postbiotics from *En. faecalis* (Nobre et al., 2022), *Lb. casei*, *Lb. rhamnosus* GG (Escamilla et al., 2012), *Lb. rhamnosus* MD-14 (Sharma and Shukla, 2020), *Lb. crispatus*, and *Lb. gasseri* have reported anti-cancer and anti-inflammatory effects in preclinical models. To the best of our knowledge, there are no available studies suggesting the impacts of postbiotics on gut and brain health or QoL in cancer survivors. The feasibility of postbiotics for gut symptoms has been established in humans, with no adverse effects reported, making it possible for future studies in cancer survivors (Tarrerias et al., 2011; Xiao et al., 2003; Liu et al., 2015; Shinkai et al., 2013).

Future studies are required to determine the mechanisms of microbiome modulators. The gut-brain-immune axis is a prominent mechanism, but more preclinical studies are necessary to improve our understanding of (1) how gut and brain dysfunctions manifest in cancer survivors and (2) how microbiome modulators may improve them. Preclinical models including *C. elegans* and rodents have wide applications for screening probiotics, prebiotics, and postbiotics and should be employed in future studies (Miller et al., 2024; Poupet et al., 2020). Cancer treatments like chemotherapy are considered systemic and have impacts not only on the gut and brain, but also on other peripheral organs like the heart and liver (Ciernikova et al., 2021; Brown et al., 2021). Therefore, future studies should study the impacts of microbiome modulators on the gut-brain-cardiac and gut-brain-liver axes in cancer survivors.

## 7 Conclusion

In conclusion, this review reports the prevalence of CI and other brain dysfunctions, as well as impaired gut health, in cancer survivors and highlights the microbiome-immune axis as a central mechanism. Long-term impairments in cognitive functions and GI health significantly hamper QoL in cancer survivors, underpinning the dire need for therapeutics. The gut microbiota has been implicated in many health conditions and its beneficial modulation may improve gut and brain functions through the gut-brain axis. Therefore, we have summarized the impacts of microbiome modulators on gut and brain dysfunctions in cancer survivors. The currently available literature is limited and the mechanisms of microbiome modulators in cancer survivors remain poorly understood. Future studies with larger sample sizes and in specialized cohorts are required for the development and widespread utilization of novel and natural precision-based microbiome modulators, including probiotics, prebiotics, synbiotics, and postbiotics, for improving QoL in cancer survivors.

## Glossary

Aβ: Amyloid-β
AD: Alzheimer’s disease
*Bf.*: *Bifidobacterium*
CAR-T: Chimeric antigen receptor t-cell therapy
*C. elegans*: *Caenorhabditis elegans*
CI: Cognitive impairment
CRC: Colorectal cancer
CRP: C-reactive protein
*Cs.*: *Clostridium*
CTCI: Cancer treatment-related cognitive impairment
CTLA-4: Cytotoxic T-lymphocyte-associated protein 4
*E.*: *Escherichia*
EEG: Electroencephalography
*En.*: *Enterococcus*
FACT: Functional Assessment of Cancer Therapy
*Fb.*: *Faecalibacterium*
FFAR2/3: Free fatty acid receptors 2 and 3
FMT: Fecal microbiota transplantation
FOLFOX-4: 5-fluorouracil, leucovorin and oxaliplatin (chemotherapy)
FOS: Fructo-oligosaccharides
GI: Gastrointestinal
GOS: Galacto-oligosaccharides
HCAR2: Hydroxycarboxylic acid receptor 2
HDAC: Histone deacetylase
hs-CRP: High-sensitivity C-reactive protein
ICIs: Immune checkpoint inhibitors
IFN-γ: Interferon-γ
IL: Interleukin
*Lb.*: *Lactobacillus*
*Lc.*: *Lactococcus*
LPS: Lipopolysaccharide
MCI: Mild cognitive impairment
MCT: Monocarboxylate transporter
MMSE: Mini-Mental State Examination
MoCA: Montreal Cognitive Assessment
MRI: Magnetic Resonance Imaging
PD-1/PD-L1: Programmed cell death-1/programmed cell death ligand-1
PHQ-9: Patient Health Questionnaire-9
QoL: Quality of life
SCFA: Short-chain fatty acid
*St.*: *Streptococcus*
TNF-α: Tumor necrosis factor-α
5-FU: 5-fluorouracil
